# Role of UPF1 in *lncRNA-HEIH* regulation for hepatocellular carcinoma therapy

**DOI:** 10.1038/s12276-024-01158-6

**Published:** 2024-02-01

**Authors:** Hyunho Cha, Minwoo Kim, Narae Ahn, Seong Dong Jeong, Elizaveta Ignatova, Sung Wook Chi, Hyeon Ho Kim, Jungwook Hwang

**Affiliations:** 1 Graduate School for Biomedical Science & Engineering, Hanyang University, Seoul, Korea; 2 Department of Health Sciences and Technology, Samsung Advanced Institute for Health Sciences and Technology, Sungkyunkwan University, Seoul, Korea; 3 Department of Life Sciences, Korea University, Seoul, Korea; 4 KU-KIST Graduate School of Converging Science and Technology, Korea University, Seoul, Korea; 5 Institute for Future Medicine, Samsung Medical Center, Seoul, Korea; 6 Hanyang Institute of Bioscience and Biotechnology, Hanyang University, Seoul, Korea

**Keywords:** Long non-coding RNAs, Liver cancer

## Abstract

UPF1, a novel posttranscriptional regulator, regulates the abundance of transcripts, including long noncoding RNAs (lncRNAs), and thus plays an important role in cell homeostasis. In this study, we revealed that UPF1 regulates the abundance of hepatocellular carcinoma upregulated EZH2-associated lncRNA *(lncRNA-HEIH*) by binding the CG-rich motif, thereby regulating hepatocellular carcinoma (HCC) tumorigenesis. UPF1-bound *lncRNA-HEIH* was susceptible to degradation mediated by UPF1 phosphorylation via SMG1 and SMG5. According to analysis of RNA-seq and public data on patients with liver cancer, the expression of *lncRNA-HEIH* increased the levels of miR-194-5p targets and was inversely correlated with miR-194-5p expression in HCC patients. Furthermore, UPF1 depletion upregulated *lncRNA-HEIH*, which acts as a decoy of miR-194-5p that targets GNA13, thereby promoting GNA13 expression and HCC proliferation. The UPF1/lncRNA-HEIH/miR-194-5p/GNA13 regulatory axis is suggested to play a crucial role in cell progression and may be a suitable target for HCC therapy.

## UPF1’s key role in regulating hepatocellular carcinoma tumorigenesis unveiled

Hepatocellular carcinoma (HCC, a type of liver cancer), has a high death rate due to limited effective treatments. Current medications often result in resistance and only prolong life by a few months. This research investigates the role of UPF1, a crucial component in a process called nonsense-mediated mRNA decay (the elimination of faulty genetic messages), in controlling the expression of a long noncoding RNA (lncRNA, a type of genetic material) known as lncRNA-HEIH. The scientists discovered that reducing UPF1 increased the level of lncRNA-HEIH, which subsequently encouraged the growth of HCC cells. Moreover, lncRNA-HEIH was found to distract a molecule named miR-194-5p, thus increasing the expression of a cancer-promoting gene called GNA13. This UPF1/lncRNA-HEIH/miR-194-5p/GNA13 regulatory pathway could potentially be targeted for therapeutic interventions in HCC. This summary was initially drafted using artificial intelligence, then revised and fact-checked by the author.

## Introduction

Hepatocellular carcinoma (HCC) has a high mortality rate because of the lack of effective treatments. Multityrosine kinase inhibitors (sorafenib and lenvatinib) and a combination of atezolizumab (anti-PD-L1 antibody) and bevacizumab (anti-VEGF antibody) are currently approved for HCC therapy^1^. However, these drugs may lead to drug resistance and extend life by only a few months. To overcome these hurdles of HCC therapy, extensive studies on HCC therapeutics have been conducted at the molecular and cellular levels. One approach is the study of noncoding RNAs (ncRNAs), including microRNAs (miRNAs) and long ncRNAs (lncRNAs). In terms of molecular functions, lncRNAs act by regulating transcription via interaction with transcription factors, translating functional peptides from small open reading frames, mediating posttranscriptional regulation via interaction with diverse RNA-binding proteins (RBPs), and acting as miRNA sponges or decoys. In HCC, for example, *lncRNA-TUG1* promotes cell growth by interacting with polycomb repressive complex 2 (PRC2), thereby decreasing KLF2 levels^2^. In terms of peptide production from lncRNAs, *lncRNA-00998*-encoded SMIM30 promotes SRC/YES1 membrane anchoring and activates the MAPK pathway^3^. *LncRNA-01138* acts as an RBP sponge, inhibiting ubiquitin/proteasome-dependent degradation by physically interacting with arginine methyltransferase 5 (PRMT5) to increase PRMT5 turnover^4^. Multiple lines of evidence suggest that lncRNAs act as decoys or sponges for miRNAs, increasing oncogene expression in HCC. *LncRNA-MCM3AP-AS1* sponges miR-194-5p, increasing the expression of FOXA1, which is also a target of miR-194-5p, and thus promotes tumor development. Additionally, *lncRNA-ATB* is known to promote EMT and invasion in HCC by competitively binding to the miR-200 family^5,6^.

UPF1, a well-known posttranscriptional regulator, regulates the abundance of lncRNAs and mRNAs. The functionality of UPF1 in the nonsense-mediated mRNA decay (NMD) process is intricately regulated by its phosphorylation status, which is mediated by the serine/threonine kinase SMG1^7^. Hyperphosphorylated UPF1 interacts with RNA decay factors such as SMG6 and SMG5/7^8^, facilitating the degradation of target RNAs. Subsequently, hyperphosphorylated UPF1 undergoes dephosphorylation by the enzyme PP2A, reverting to its hypophosphorylated form, hypo-UPF1^9^. This hypo-UPF1 pool is recycled for subsequent rounds of NMD. UPF1 recognizes premature termination codon (PTC)-containing transcripts with various NMD factors or initiates the degradation of long 3’UTR-containing transcripts with the help of miRNAs in the regulation of mRNA expression^10–13^. Notably, UV cross-linking RNA immunoprecipitation sequencing (CLIP-seq) demonstrated that UPF1 was also associated with a variety of lncRNAs^14^, indicating that UPF1 has the potential to regulate cell fate by reducing the abundance of lncRNAs, although the detailed mechanism by which UPF1 degrades lncRNAs remains unknown. The binding of UPF1 to *lncRNA-UCA1* was found to reduce the abundance of *lncRNA-UCA1*, inhibiting HCC growth^15^. Moreover, the interaction of UPF1 and *lncRNA-SNAI3-AS1* regulates Smad7 expression and activates TGF-β/Smad signaling^16^. In addition to its role in lncRNA regulation, UPF1 and its variants increase the stability of the tumor suppressor protein DUSP1, resulting in phosphorylation of protein p53 and inhibition of HCC growth^17^. One of the lncRNAs, *lncRNA-HEIH* (hepatocellular carcinoma upregulated EZH2-associated lncRNA), inhibits the expression of cell cycle regulatory genes via histone methylation mediated by enhancer of zeste homolog 2 (EZH2), a component of the PRC2 complex, and facilitates cell growth^18^. Other studies have revealed that *lncRNA-HEIH* activates the PI3K/AKT pathway by sponging miR-98, thereby increasing resistance to sorafenib^19^.

Here, we investigated the role of UPF1 in HCC. Transcriptome analysis in UPF1-depleted cell lines showed that *lncRNA-HEIH* was commonly upregulated. Consistent with the effects of UPF1 depletion (enhanced HCC cell growth), exogenous *lncRNA-HEIH* promoted HCC tumorigenesis. Analysis of the public UPF1 CLIP-seq dataset and biochemical assays revealed that UPF1 binds to the double-stranded region in *lncRNA-HEIH* and that its degradation is dependent on UPF1 phosphorylation and SMG5. *LncRNA-HEIH* acts as a decoy of miR-194-5p, which targets the oncogene GNA13. Our findings demonstrate that UPF1 regulates *lncRNA-HEIH* levels in HCC, and this lncRNA recruits miR-194-5p and regulates GNA13 expression, ultimately demonstrating therapeutic potential in HCC.

## Materials and methods

### Cell culture and transfection

Three HCC cell lines (Huh7, SNU-354, and PLC/PRF/5), HEK293T and HeLa cells were maintained in Dulbecco’s modified Eagle’s medium (DMEM) supplemented with 10% fetal bovine serum (FBS) and 1% penicillin/streptomycin. To deplete or overexpress the target gene, the cells were transfected with siRNA, antisense oligonucleotide (ASO), and DNA plasmid using Lipofectamine 3000 (Invitrogen, #L3000015). All cells were confirmed to be free of mycoplasma contamination by using a cell culture contamination detection kit (InvivoGen, #rep-mys-10). Target siRNA, ASO and guide RNA (gRNA) for CasRX sequences are listed in Supplementary Tables 1, 3, and 5.

### Plasmid construction

We constructed pcDNA3.1-HEIH to overexpress HEIH by digesting a pcDNA3.1 plasmid with EcoRI and XbaI and ligating the digested vector fragment to PCR-amplified fragments that were also digested with EcoRI and XbaI. The PCR products were generated by amplification of genomic DNA (gDNA) from Huh7 cells and two primers, HEIH-EcoRI-F and HEIH-XbaI-R. Similarly, pcDNA3.1-HEIH-S1, -S2, and -S3 PCR fragments were generated using pcDNA3.1-HEIH, HEIH-EcoRI-F and HEIH-S1-XbaI-R for lncRNA-HEIH-S1, HEIH-S2-EcoRI-F and HEIH-S2-XbaI-R for lncRNA-HEIH-S2, and HEIH-S3-EcoRI-F and HEIH-XbaI-R for lncRNA-HEIH-S3, respectively.

To construct pmirGLO-UPF1-bs-HEIH WT, a pmirGLO plasmid was digested with XbaI and NotI and ligated to PCR-amplified fragments that were also digested with the same digestion enzyme. PCR products were generated by amplifying pcDNA3.1-HEIH using two primers, UPF1-bs-WT-XbaI-F and UPF1-bs-WT-NotI-R. For the pmirGLO-UPF1-bs-HEIH Mut construct, the same digested vector was ligated to the PCR fragment generated by annealing the two primers UPF1-bs-Mut-F and UPF1-bs-Mut-R and amplified using the two primers UPF1-bs-WT-XbaI-F and UPF1-bs-WT-NotI-R.

To construct mutated UPF1 binding sites (bs) in pcDNA3.1-HEIH, the pcDNA3.1 plasmid digested with EcoRV was infused with three PCR products named Fragments 1, 2, and 3. Fragments 1 and 3 were generated by amplifying pcDNA3.1-HEIH with the two primer pairs listed in Supplementary Table 2. Fragment 2 was generated by amplifying pmirGLO-UPF1-bs-HEIH mut. For UPF1 binding mutated sites in lncRNA-HEIH-S2, the pcDNA3.1 plasmid was digested by HindIII, and NotI was ligated to PCR-amplified fragments that were also digested with the same digestion enzyme. The PCR product was generated by amplifying pcDNA3.1-HEIH Mut using primers HEIH-S2-UPF1-bs-Mut-HindIII-F and HEIH-S2-UPF1-bs-Mut-notI-R.

We constructed pmirGLO-GNA13-3’UTR WT and pmirGLO-GNA13-3’UTR Mut by digesting a pmirGLO plasmid with SacI and XbaI and infusing the digested vector fragment into PCR-amplified fragments. The PCR products were generated by annealing the two pairs of synthesized oligos for WT and Mut, GNA13-3’UTR-5’ and GNA13-3’UTR WT-3’ for WT, GNA13-3’UTR-5’ and GNA13-3’UTR Mut-3’ for Mut, and amplified using the two primer pairs for WT and Mut, GNA13-3’UTR-F and GNA13-3’UTR WT-R for WT, and GNA13-3’UTR-F and GNA13-3’UTR Mut-R for Mut.

We constructed pmirGLO-HEIH-miR-194-5p WT and pmirGLO-HEIH-miR-194-5p Mut by digesting the pmirGLO plasmid with SacI and XbaI and infusing the digested vector fragment into PCR-amplified fragments. The PCR products were generated by annealing the two primer pairs for WT and Mut, HEIH-miR-194-5p-5’ and HEIH-miR-194-5p WT-3’ for WT, HEIH-miR-194-5p-5’ and HEIH-miR-194-5p Mut-3’ for Mut, and amplified using the two primer pairs for WT and Mut, HEIH-miR-194-5p-F and HEIH-miR-194-5p WT-R for WT, HEIH-miR-194-5p-F, and HEIH-miR-194-5p Mut-R for Mut.

We constructed pcDNA3.1-GNA13 by digesting the pcDNA3.1 plasmid with EcoRI and XhoI and infusing the digested vector fragment into PCR-amplified fragments. The PCR products were generated by amplification of cDNA from total RNA of Huh7 cells using two primers, GNA13-F and GNA13-R.

To construct CasRX-gRNA targeting lncRNA-HEIH, we digested the pXR003 plasmid with BbsI and synthesized Fragment gRNA#1 or #2 sequences that were infused into the digested vector.

We constructed pcDNA3.1-luciferase by digesting the pcDNA3.1 plasmid with BamHI and EcoRI and infusing the digested vector fragment into PCR-amplified fragments. The PCR products were generated by amplification of the pmirGLO vector using two primers, luciferase-F and luciferase-R. All primers used are listed in Supplementary Table 2.

### Western blotting (WB)

To detect specific proteins, proteins in total cell lysates were eluted with SDS and β-mercaptoethanol. Proteins were then separated on gels containing various percentages of polyacrylamide and transferred to nitrocellulose membranes. WB was performed using antibodies specific to the following proteins: UPF1 (Cell Signaling, #12040 S), calnexin (Cell Signaling, #2679), β-actin (Sigma, #A2228), FLAG (Sigma, #F3165), GST (GE Healthcare, #27-4577), EZH2 (Cell Signaling, #5246 S), and GNA13 (Thermo Fisher, #PA5-117026).

### RT‒qPCR

Total RNA was extracted using TRIzol reagent (Invitrogen, #15596026). To remove exogenous and endogenous DNA, extracted RNA was treated with RNA qualified (RQ) DNase I (Promega, #M6101). cDNA was synthesized using reverse transcriptase (RTase) (Thermo Fisher, #EP0441) with random hexamer primers (Macrogen). RT‒qPCR was performed using the primers listed in Supplementary Table 4.

### Calculation of NMD efficiency

The relative NMD efficiency was obtained using the following formula: fold change of NMD = Test NMD/Control NMD, where NMD = Relative Ter mRNA/Relative Norm mRNA. NMD was obtained from the relative PTC-containing mRNA (Ter) normalized to the relative PTC-free mRNA (Norm), in which both PTC-containing and PTC-free mRNA were normalized to MUP mRNA as a reference.

### Migration and invasion assay

To evaluate cell migration, monolayers of HCC cells transfected with lncRNA-HEIH plasmid and in vitro transcribed RNA were scratched with a sharp pipette tip, and the area of migration was measured using ImageJ software. Similarly, transfected cells were resuspended in serum-free medium and seeded on a Matrigel-coated membrane (Corning, #354480) in an invasion chamber insert. The cells that had penetrated the membrane after 24 h of incubation were visualized using hematoxylin and eosin staining.

### Statistical analysis

Unpaired Student’s *t* tests were used to calculate the p values in the RT‒qPCR analyses. Different *p* values < 0.05 (*), 0.01 (**), 0.001 (***), or ns (not significant), as indicated in the figure, were considered statistically significant by GraphPad software. The mean ± SEM values were calculated from independent experiments. The Wald test was used to calculate *p* values for the volcano plot.

### ASO pull-down assay

To observe lncRNA-HEIH-interacting proteins and miRNA, biotinylated ASO against lncRNA-HEIH was incubated with the indicated Huh7 cell lysate, and ASO was pulled down against streptavidin-conjugated beads. The proteins and miRNAs interacting with lncRNA-HEIH were analyzed by WB and RT‒qPCR, respectively. The ASO sequence is listed in Supplementary Table 3.

### Immunoprecipitation (IP)

To determine the UPF1 binding sites in lncRNA-HEIH, we performed IP using a UPF1 antibody in Huh7 cells. Huh7 cells were transiently transfected with the indicated DNA plasmids expressing WT or mutant pcDNA3.1-HEIH or HEIH-S2, as described previously^12^. Huh7 cell lysates that were transfected with FLAG-AGO2 were employed for IP using anti-FLAG beads (Sigma, #A2220). WB and RT‒qPCR were used to examine coimmunoprecipitated proteins and RNA, respectively.

### In vitro transcription and binding assay

RNA pull-down assays were performed using biotinylated WT or Mut S2 lncRNA-HEIH. Briefly, the biotinylated lncRNA-HEIH S2 RNA was transcribed with a Biotin RNA Labeling Mix Kit (Roche, #11685597910) and T7 RNA polymerase (TaKaRa, #2540 A). Three pmole of transcribed RNA was applied for further pull-down assays. Meanwhile, the HEK293T cell lysates that were transfected with GST-MYC-UPF1 were purified with GST-antibody conjugated beads (GE Healthcare, #17-0756) in the presence of a nuclease mixture (DNase and RNase). One pmole of GST-MYC-UPF1 was incubated with three pmoles of in vitro transcribed RNA for 2 h at 4 °C. Afterward, 40 μl of streptavidin-conjugated magnetic beads (NEB, #S1420S) was added for another hour of incubation at room temperature. The beads were washed ten times and boiled with 1×SDS loading buffer for 10 min at 95 °C. The RNA-bound proteins and RNA were analyzed by WB and RT-sqPCR, respectively.

To overexpress RNA in Huh7 cells, RNA was transcribed in vitro. LncRNA-HEIH and luciferase in pcDNA3.1 were transcribed by T7 polymerase (TaKaRa, #2540 A) in the presence of methylpseudouridine (Jena bioscience, #NU-890S) to evade the innate immune system.

### Cell fractionation

Huh7 cells that were UPF1-depleted or UPF1-overexpressing were separated into nuclear and cytoplasmic fractions using NE-PER™ Nuclear and Cytoplasmic Extraction Reagents (Thermo Fisher, #78833). Briefly, cell lysates were gently lysed and centrifuged. The supernatant was considered to be the cytoplasmic fraction, and the pellet was resuspended with high-salt buffer and centrifuged; the resulting supernatant was considered to be the nuclear fraction.

### Generation of UPF1 KD cell line by CRISPR/Cas9

HeLa cells were transfected with the Cas9-puro-2A-RFP vector and two sgRNAs, 5’-AGAAGACACCTATTACACGA-3’ and 5’-AAAGGCAAGACTGGTCGTGG-3’ targeting exon 2 and exon 22 of UPF1, respectively^20^. The two sgRNAs were cloned and inserted into pRG2-Ext(Gx19)^20^. Puromycin was used to select the cells. WB was used to measure the levels of UPF1 in selected cells after colonization of each cell in 96-well plates. The genomic UPF1 sequence in two selected clones, clone 9 and clone 41, was confirmed by Sanger sequencing.

### RNA-seq and data analysis

The total RNA concentration was measured using Quant-IT RiboGreen (Invitrogen). The RNA integrity number (RIN) was measured on a TapeStation RNA ScreenTape (Agilent Technologies). Only high-quality RNA preparations with an RIN greater than 7.0 were used for RNA library construction. A library was independently prepared with 1 μg of total RNA from each sample using the Illumina TruSeq Stranded mRNA Sample Prep Kit (Illumina). The first step in the workflow involves purifying the poly-A-containing mRNA molecules using poly‐T‐attached magnetic beads. Following purification, mRNA was fragmented into small pieces using divalent cations at high temperatures. The cleaved RNA fragments were copied into first-strand cDNA using SuperScript II reverse transcriptase (Invitrogen) and random primers. This was followed by second-strand cDNA synthesis using DNA polymerase I, RNase H, and dUTP. These cDNA fragments then go through an end repair process, the addition of a single “A” base, and then ligation of the adapters. The products were purified and enriched by PCR to create the final cDNA library. Indexed libraries were then submitted to Illumina NovaSeq (Illumina), and paired-end (2 × 100 bp) sequencing was performed by Macrogen Inc. All raw datasets were deposited in the NCBI Gene Expression Omnibus (GEO; https://www.ncbi.nlm.nih.gov/geo) under accession number GSE216667.

## Results

### *LncRNA-HEIH* regulates HCC tumorigenesis

**Fig. 1:**
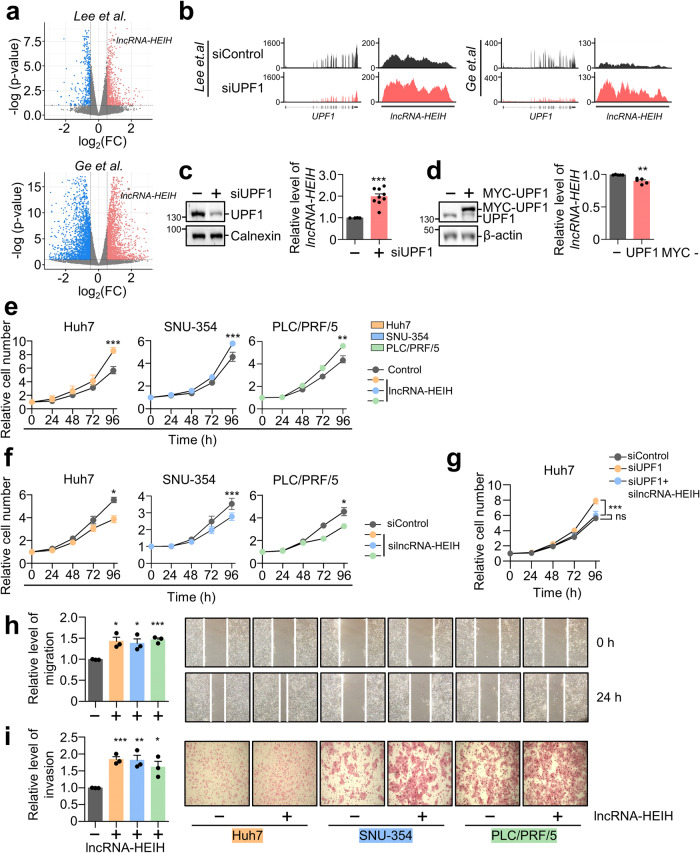
*LncRNA-HEIH* regulates HCC tumorigenesis. **a** Volcano plot showing the log2-fold change in expression between (i) UPF1-depleted Huh7 cells and control Huh7 cells performed by Lee et al.^17^ and (ii) UPF1-depleted HEK-293T cells and control HEK-293T cells performed by Ge et al.^23^. **b** Integrative Genomics Viewer (IGV) display of the increased level of *lncRNA-HEIH* upon UPF1 depletion from RNA sequencing in (**a**). The levels of *lncRNA-HEIH* were measured by RT‒qPCR from UPF1-depleted (**c**) or MYC-UPF1-overexpressing (**d**) Huh7 cells. WB was performed to evaluate the level of endogenous or exogenous UPF1. The indicated cell lines transfected with lncRNA-HEIH (**e**) or silncRNA-HEIH (**f**) were used to measure cell growth over a 96 h period. The relative level of mRNA was normalized to that of *GAPDH* mRNA in (**c**, **d**). **g** Similar to (**f**), however, UPF1 was depleted in the presence or absence of lncRNA-HEIH. Cell migration assays (**h**) and invasion assays (**i**) were performed using the indicated HCC cell lines transfected with lncRNA-HEIH. Relative migration and invasion rates were quantified. *p ≤ 0.05; **p ≤ 0.01; ***p ≤ 0.001; ns not significant.

The function of UPF1 in HCC has been extensively studied, and it has been revealed that UPF1 overexpression inhibits HCC growth^17,21,22^. As it is known that overexpression of UPF1 does not alter the level of NMD targets in Huh7 cells^17^, we sought to understand how UPF1 modulates HCC tumorigenesis as a posttranscriptional regulator. In contrast to UPF1 overexpression, which inhibited the growth of various HCC cell lines, UPF1 depletion facilitated the growth of HCC cell lines (Huh7, SNU-354, and PLC/PRF/5; Huh7 and PLC/PRF/5 had a p53 mutation, but SNU-354 had wild-type p53) (Supplementary Fig. 1a). This observation prompted us to investigate the potential transcripts regulated by UPF1, and we performed transcriptome analysis using RNA-seq data from UPF1-depleted Huh7 cells and UPF1-depleted HEK-293T cells (Fig. 1a, b)^17,23^. Depletion of UPF1 in cell lines increased the stability of many transcripts; we selected *linc00848* (also known as *lncRNA-HEIH*), which was commonly upregulated among the cell lines and promotes HCC growth^18^. The RNA-seq results were validated by quantitative real-time PCR (RT‒qPCR) using lysates of UPF1-depleted or UPF1-overexpressing cells (Fig. 1c, d).

Increases and decreases in the growth rates of HCC cell lines were confirmed in l*ncRNA-HEIH* overexpression and depletion experiments, respectively (Fig. 1e, f and Supplementary Fig. 1b–e). Moreover, depletion of *lncRNA-HEIH* reversed the effects of UPF1 knockdown on cell growth (Fig. 1g). To rule out the possibility that siRNA-mediated depletion may result in off-target effects, we generated UPF1 knockdown (KD) HeLa cells by the CRISPR/Cas9 method (Supplementary Fig. 2a). UPF1 protein and mRNA levels were significantly reduced in UPF1 KD cells, which increased the level of PTC-containing NMD reporters, indicating that UPF1 KD cells were functionally null for the posttranscriptional regulator (Supplementary Fig. 2b–d). Notably, *lncRNA-HEIH* was also significantly upregulated in UPF1 KD cell lines (Supplementary Fig. 2e, f). Given the influence of *lncRNA-HEIH* on HCC cell growth, we determined the migration and metastatic potential of the three different HCC cell lines (Fig. 1h, i, Supplementary Fig. 1f, g). Consistent with the effects of *lncRNA-HEIH* overexpression on HCC cell growth, *lncRNA-HEIH* overexpression efficiently increased the migration and invasion abilities of all three HCC cell lines. The GEPIA database analysis revealed that *lncRNA-HEIH* expression was significantly higher in liver cancer tissues than in normal liver tissues, and HCC patients with low *lncRNA-HEIH* expression had higher survival and disease-free survival rates than those with high expression (Supplementary Fig. 3a–c). Finally, the stability of *lncRNA-HEIH* increased in the UPF1-depletion condition, suggesting that *lncRNA-HEIH* was posttranscriptionally regulated by UPF1 (Supplementary Fig. 3d). These findings show that UPF1 depletion promoted HCC tumorigenesis by upregulating *lncRNA-HEIH*.

### UPF1 regulates *lncRNA-HEIH* by binding

**Fig. 2:**
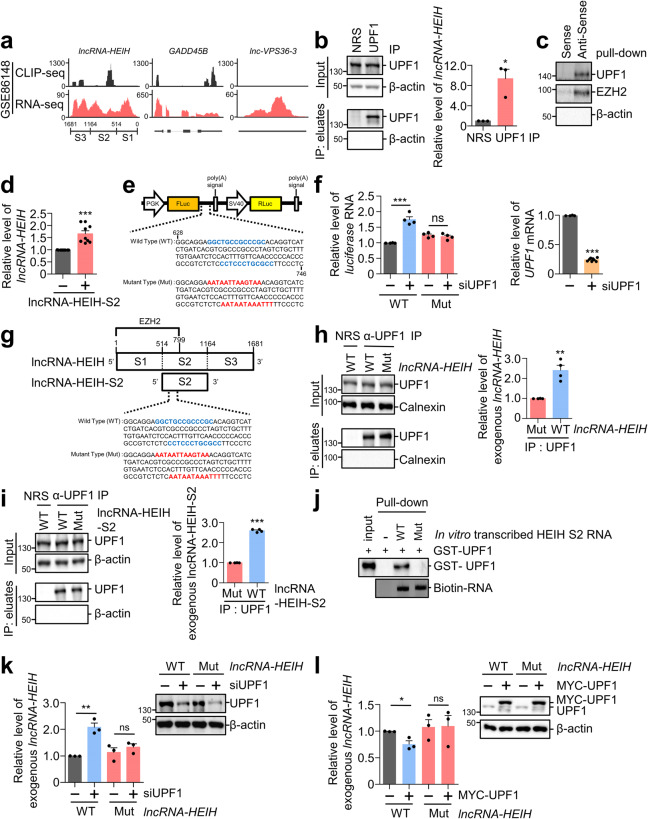
UPF1 posttranscriptionally regulates *lncRNA-HEIH* expression. **a** UPF1 binding sites on *lncRNA-HEIH*, positive (*GADD45B*), and negative control (*lnc-VPS36-3*) using public UPF1 CLIP-seq datasets were visualized. S1, S2, and S3 in lncRNA-HEIH indicate the relative position. **b** UPF1-bound *lncRNA-HEIH* in Huh7 cells was analyzed by immunoprecipitation (IP) using an anti-UPF1 antibody. IP eluates were analyzed by WB and RT‒qPCR analysis. **c** Endogenous *lncRNA-HEIH* in Huh7 cells was pulled down by antisense oligonucleotide (ASO) or sense oligonucleotide as a negative control. WB was performed to evaluate *lncRNA-HEIH*-bound proteins. **d** The relative *lncRNA-HEIH* levels were measured by RT‒qPCR after transfection of the S2 region of lncRNA-HEIH (lncRNA-HEIH-S2) in Huh7 cells. **e** Schematic representation of a dual-luciferase construct containing putative WT or Mut UPF1 binding sites from 628 to 746 in *lncRNA-HEIH*. The two putative wild-type (WT) UPF1-binding sequences (blue bold) in the S2 region, which were mutated to an AT-rich sequence (red bold, Mut), are indicated. **f** WT or Mut UPF1 binding site-expressing reporter plasmids in (**e**) were cotransfected with siUPF1 or control siRNA in Huh7 cells. The relative amount of *FLuc* mRNA normalized to the level of *RLuc* mRNA was measured by RT‒qPCR. **g** Schematic representation of lncRNA-HEIH. The full-length lncRNA-HEIH was divided into three regions (S1, S2, and S3). The indicated sequences (WT and Mut) were the same as in (**e**). **h** Huh7 cell lysates that were cotransfected with WT or Mut lncRNA-HEIH and MUP plasmid as a transfection control were employed for IP against anti-UPF1 antibody. WB and RT‒qPCR were performed to evaluate the indicated proteins and coimmunoprecipitated *lncRNA-HEIH* with UPF1. NRS; normal rabbit serum. **i** Similar to (**h**), however, samples were from cells transfected with WT or Mut lncRNA-HEIH-S2 plasmid. **j** Purified GST-UPF1 and in vitro transcribed WT or Mut lncRNA-HEIH-S2 were employed for the in vitro biotinylated RNA binding assay. RNA-bound GST-UPF1 and biotinylated RNA were observed by WB and semi RT‒qPCR (RT-sqPCR), respectively. WT or Mut lncRNA-HEIH plasmids were transfected into UPF1-depleted (**k**) or UPF1-overexpressing (**l**) Huh7 cells. The relative level of mRNA was normalized to that of *GAPDH* mRNA (**d**, **f**) or MUP mRNA (**h**, **i**). *p ≤ 0.05; **p ≤ 0.01; ***p ≤ 0.001; ns not significant.

UPF1, a double-stranded RNA-binding protein, initiates posttranscriptional regulation by associating with its target transcript. We first determined UPF1 binding sites on *lncRNA-HEIH* by analyzing public UPF1 CLIP-seq, which indicated that UPF1 binding sites were enriched in the middle of lncRNA-HEIH, S2 region (Fig. 2a)^24^. These results were confirmed by UPF1 IP and RNA pull-down using lncRNA-HEIH antisense oligonucleotides (ASOs) in Huh7 cells (Fig. 2b, c). UPF1 interacts with *lncRNA-HEIH*, as demonstrated by RT‒qPCR using UPF1 IP eluates and WB using *lncRNA-HEIH* pull-down eluates. To determine UPF1 binding to *lncRNA-HEIH*, we divided lncRNA-HEIH into three domains based on length (S1, S2, and S3), with the lncRNA-HEIH-S2 region containing the putative UPF1 binding sites in CLIP-seq. First, we hypothesized that exogenous lncRNA-HEIH-S2 would compete with endogenous *lncRNA-HEIH* for UPF1 binding, thereby increasing *lncRNA-HEIH* levels. Indeed, overexpression of lncRNA-HEIH-S2 increased the expression of endogenous *lncRNA-HEIH* by up to 1.5-fold compared to that of lncRNA-HEIH-S1 and lncRNA-HEIH-S3 (Fig. 2d and Supplementary Fig. 4a). As UPF1 prefers to bind the CUG-centered CG-rich motif in double-stranded RNA (dsRNA)^25^, we mutated two CG-rich motifs in the S2 region to AT-rich regions in lncRNA-HEIH and lncRNA-HEIH-S2, which were predicted to be single-stranded RNA (ssRNA) regions (Supplementary Fig. 4b). We then performed a bicistronic reporter assay in the presence or absence of UPF1, where we inserted the wild type (WT) or mutant (Mut) UPF1 binding sites of lncRNA-HEIH into the 3’UTR of FLuc, as depicted in Fig. 2e. RT‒qPCR results showed that UPF1 depletion increased the level of reporter transcripts containing WT UPF1 binding sites but not Mut sites (Fig. 2f). To confirm the reporter assay results, we generated mutation sites in the whole lncRNA-HEIH or lncRNA-HEIH-S2 (Fig. 2g). UPF1 IP and RT‒qPCR analyses demonstrated that UPF1 preferentially binds to WT *lncRNA-HEIH* (Fig. 2h). These observations were validated with lncRNA-HEIH-S2 (Fig. 2i). In addition, we performed an RNA pull-down assay using purified GST-MYC-UPF1 and in vitro biotinylated lncRNA-HEIH-S2, indicating that UPF1 specifically binds to WT lncRNA-HEIH-S2 (Fig. 2j, Supplementary Fig. 4c, d). Depletion of UPF1 consistently increased the levels of WT *lncRNA-HEIH* but not Mut *lncRNA-HEIH* (Fig. 2k). In contrast, overexpression of UPF1 reduced the levels of WT *lncRNA-HEIH* (Fig. 2l). Overall, these findings suggest that UPF1 regulates the expression of *lncRNA-HEIH* by binding.

### UPF1 may regulate *lncRNA-HEIH* via SMG1 and SMG5

**Fig. 3:**
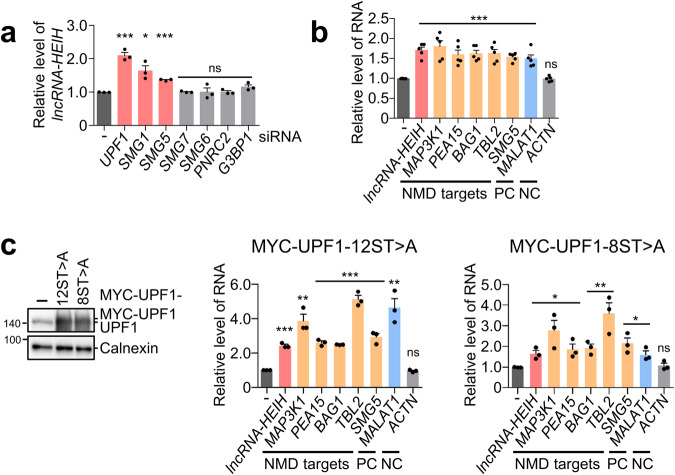
UPF1 phosphorylation is necessary for the regulation of *lncRNA-HEIH* via SMG5. **a** The indicated NMD factors were depleted by siRNA in Huh7 cells. **b** Huh7 cells treated with 100 nM okadaic acid were employed for RT‒qPCR. **c** The UPF1 variants (MYC-UPF1-12ST > A and MYC-UPF1-8ST > A), which contained twelve or eight serines/threonine to alanine mutations, increased the level of unphosphorylated UPF1. The level of target mRNA detected by RT‒qPCR was normalized to that of *GAPDH* mRNA (**a**–**c**). PC positive control, NC negative control. *p ≤ 0.05; **p ≤ 0.01; ***p ≤ 0.001; ns not significant.

UPF1 is a key regulator that removes the target transcript in a phosphorylation state by SMG1^9^. Phosphorylated UPF1 triggers mRNA decay upon binding with its partners, including SMG5, SMG6, SMG7, or PNRC2. Among the UPF1-mediated decay factors, *lncRNA-HEIH* was upregulated when SMG1 and SMG5 were depleted (Fig. 3a). To assess whether phosphorylated UPF1 is involved in regulating *lncRNA-HEIH*, Huh7 cells were treated with okadaic acid (OA), a PP2A inhibitor that increased phosphorylated UPF1 levels while preventing UPF1 reuse and inhibiting NMD. RT‒qPCR analysis demonstrated that the levels of *lncRNA-HEIH* and NMD targets were upregulated by OA treatment, suggesting that UPF1 recycling is needed for regulating *lncRNA-HEIH* stability (Fig. 3b). Then, we employed UPF1 variants that cannot be phosphorylated by SMG1 (MYC-UPF1-12ST > A and MYC-UPF1-8ST > A)^26^, and the results indicated that the abundance of *lncRNA-HEIH* was dependent on UPF1 phosphorylation status (Fig. 3c). Together, these results indicate that UPF1 phosphorylation by SMG1 and its binding partner, SMG5, are potentially involved in *lncRNA-HEIH* regulation.

### *LncRNA-HEIH* acts as a decoy of miR-194-5p

**Fig. 4:**
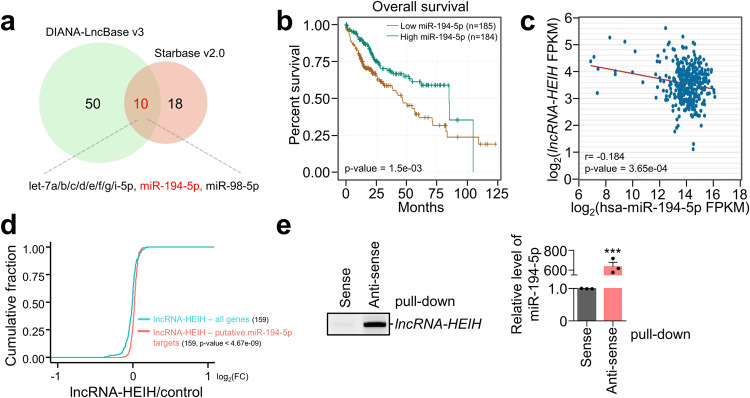
*LncRNA-HEIH* acts as a decoy of miR-194-5p. **a** The putative *lncRNA-HEIH*-associated miRNAs were predicted by the indicated two independent algorithms. **b** Kaplan‒Meier curves displaying the overall survival of patients with low miR-194-5p vs. high miR-194-5p expression in the TCGA database from the starBase V2.0 platform. **c** *LncRNA-HEIH* expression was inversely correlated with miR-194-5p expression in HCC tissues based on the TCGA database from the starBase V2.0 platform. FPKM; fragments per kilobase per million mapped reads. **d** Cumulative log2-fold changes in expression are shown as CDF plots using the putative miR-194-5p targets, which have over 10 FPKM in RNA-seq using *lncRNA-HEIH*-overexpressing Huh7 cells. **e** *LncRNA-HEIH*-associated miR-194-5p using ASO was quantified by RT-sqPCR (right panel). The amount of pulled down *lncRNA-HEIH* was normalized to the amount of spiked bacterial *LacZ* mRNA and visualized by RT‒PCR (left panel). ***p ≤ 0.001.

Cytoplasmic lncRNAs absorb cellular miRNAs, thereby increasing the number of miRNA targets^27–31^. Because *lncRNA-HEIH* is predicted to be localized in the cytoplasm (https://lncatlas.crg.eu/)^32^, we first examined the localization of *lncRNA-HEIH* in Huh7 cells by cell fractionation and RT‒qPCR (Supplementary Fig. 5a). RT‒qPCR revealed that *lncRNA-HEIH* was equally distributed in the cytoplasm and nucleus, while small nuclear RNA (*U6 snRNA*) and ribosomal RNA 28S (*28S rRNA*) were primarily confined to the nucleus and the cytoplasm, respectively. Remarkably, changes in *lncRNA-HEIH* levels resulting from UPF1 depletion and overexpression predominantly occurred within the cytoplasmic compartment, suggesting a putative cytoplasmic role for UPF1-regulated *lncRNA-HEIH* (Supplementary Fig. 5b, c). To identify the target miRNAs that could interact with *lncRNA-HEIH*, we screened two databases and selected the common putative miRNA-interacting *lncRNA-HEIH* (Fig. 4a). As *lncRNA-HEIH* promotes oncogenic functions, including cell proliferation, migration, and invasion (Fig. 1), we hypothesized that it has the potential to absorb or act as a decoy of tumor-suppressive miRNAs. Among putative miRNAs interacting with *lncRNA-HEIH*, miR-194-5p expression was inversely correlated with the survival rate of patients with HCC (Fig. 4b). In addition, TCGA dataset analysis revealed a significant inverse correlation between *lncRNA-HEIH* and miR-194-5p expression in patients with HCC (Fig. 4c). The putative miR-194-5p targets were more derepressed by *lncRNA-HEIH* overexpression than non-targets in RNA-seq using Huh7 cells overexpressing *lncRNA-HEIH* (Fig. 4d and Supplementary Fig. 5d). In contrast with miR-194-5p, let-7 family did not significantly change the abundance of their targets in the *lncRNA-HEIH* overexpression and was not correlated with the survival rate of HCC patients (Supplementary Fig. 5e, f). To determine whether miR-194-5p binds to *lncRNA-HEIH*, we performed an RNA pull-down assay using ASOs and found that miR-194-5p was enriched in the *lncRNA-HEIH* pull-down eluates (Fig. 4e). These results indicate that l*ncRNA-HEIH* acts as a decoy of miR-194-5p, increasing the expression of miR-194-5p targets in HCC.

**Fig. 5:**
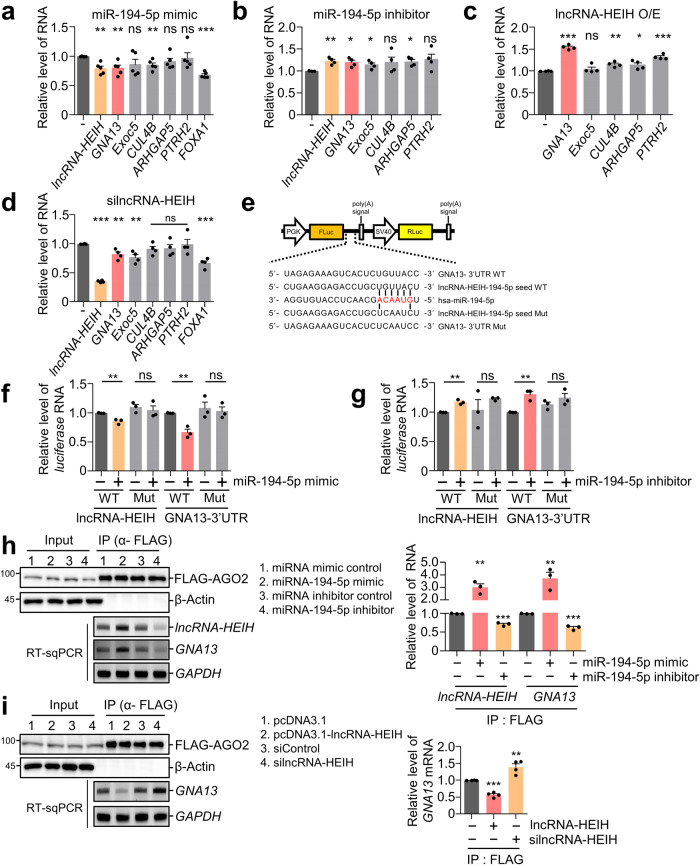
miR-194-5p regulates the expression of *lncRNA-HEIH* and *GNA13* mRNA. Huh7 cells were transfected with miR-194-5p mimic (**a**), miR-194-5p inhibitor (**b**), lncRNA-HEIH (**c**) or silncRNA-HEIH (**d**). **e** Schematic representation of a bicistronic reporter plasmid containing *lncRNA-HEIH* or *GNA13* mRNA 3’UTR that had a putative miR-194-5p binding site (WT) or mutated binding sites (Mut). **f** miR-194-5p mimic or control miRNA mimic was cotransfected with the indicated reporter plasmid in Huh7 cells. **g** Similar to (**f**), but instead of the miR-194-5p mimic, the miR-194-5p inhibitor was used for transfection. RT‒qPCR was performed to quantify the relative amount of *FLuc* mRNA normalized to the level of *RLuc* mRNA. **h** FLAG-tagged AGO2 was transfected into Huh7 cells in the presence of miR-194-5p mimic or inhibitor, and IP was performed using anti-FLAG beads. WB, RT-sqPCR, and RT‒qPCR were employed to evaluate IP eluates and the target RNA. **i** Similar to (**h**), but *lncRNA-HEIH* was overexpressed or depleted. The level of target mRNA was normalized to that of *GAPDH* mRNA (**a**–**d**, **h** and **i**). *p ≤ 0.05; **p ≤ 0.01; ***p ≤ 0.001; ns not significant.

These observations led us to investigate the targets of miR-194-5p, which are involved in oncogenesis. We selected five targets (*GNA13, EXOC5, CUL4B, ARHGAP5*, and *PTRH2*) based on the following criteria: (i) the transcript is upregulated by *lncRNA-HEIH* overexpression in RNA-seq analysis (Supplementary Fig. 6a), (ii) the transcript contains the binding sites for miR-194-5p in its 3’UTR, and (iii) the transcript is involved in tumorigenesis. The level of putative miR-194-5p targets was then determined in Huh7 cells transfected with miR-194-5p mimic or miR-194-5p inhibitor (Fig. 5a, b). RT‒qPCR analysis demonstrated that only *lncRNA-HEIH* and *GNA13* were downregulated by the miR-194-5p mimic and upregulated by the inhibitor, and *FOXA1* served as a positive control^5^. Furthermore, *lncRNA-HEIH* overexpression and depletion increased and decreased the *GNA13* levels, respectively (Fig. 5c, d). A simple interpretation of these results is that *lncRNA-HEIH* acts as a decoy of miR-194-5p, thereby upregulating *GNA13*, which acts as an oncogenic protein^33–35^ and is conserved in primates (Supplementary Fig. 6b). To ensure that *lncRNA-HEIH* and *GNA13* are miR-194-5p targets, we generated bicistronic reporter constructs with either WT or mutated miR-194-5p binding sites in the lncRNA-HEIH or 3’UTR of GNA13 (Fig. 5e). The exogenous miR-194-5p mimic reduced the expression of WT miR-194-5p binding sites containing lncRNA-HEIH and the GNA13-3’UTR but had no effect on mutant binding sites (Fig. 5f). Consistent with this, the introduction of the miR-194-5p inhibitor upregulated the abundance of WT miR-194-5p binding site-containing lncRNA-HEIH and the GNA13-3’UTR (Fig. 5g). To examine the interaction between lncRNA-HEIH/GNA13 and miR-194-5p, an Argonaute 2 (AGO2) IP experiment was performed. The introduction of miR-194-5p in Huh7 cells increased the enrichment of *lncRNA-HEIH* and *GNA13* in AGO2 IP eluates compared to that upon treatment with the control miRNA mimic. Consistently, the introduction of the miR-194-5p inhibitor reduced the levels of AGO2-bound *lncRNA-HEIH* and *GNA13* (Fig. 5h). The overexpression and depletion of *lncRNA-HEIH* reduced and increased the level of AGO2-bound *GNA13*, respectively (Fig. 5i), suggesting that *lncRNA-HEIH* competes with *GNA13* for miR-194-5p binding. The results from the biochemical assays indicate that miR-194-5p targets *lncRNA-HEIH* and *GNA13*.

### *LncRNA-HEIH* regulates GNA13 abundance

**Fig. 6:**
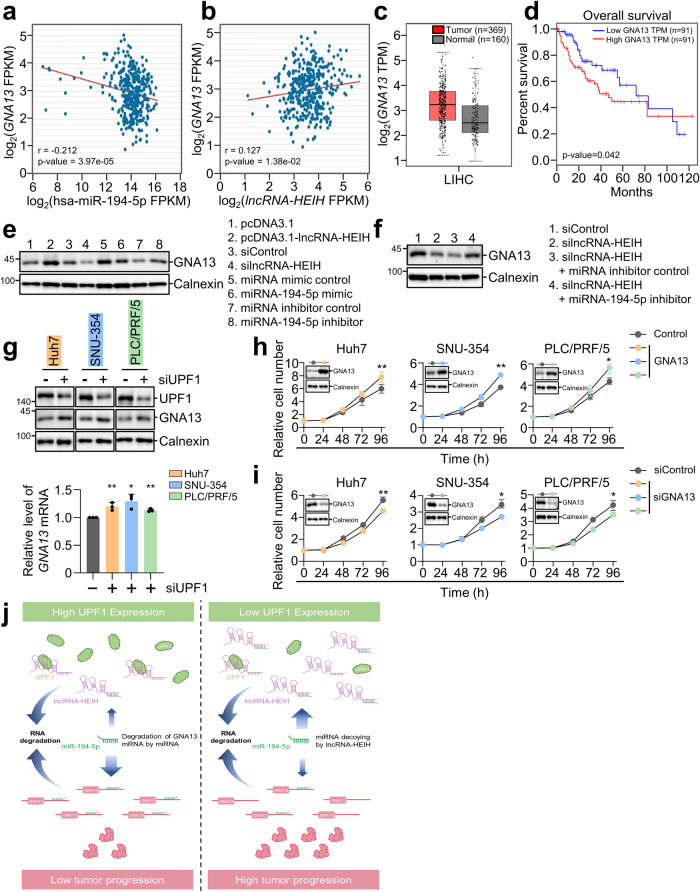
LncRNA-HEIH/miR-194-5p/GNA13 axis regulates HCC proliferation. *GNA13* expression was inversely correlated and correlated with miR-194-5p (**a**) and lncRNA-HEIH (**b**) expression, respectively, in HCC tissues based on the TCGA database from starBase V2.0 platform. **c** Meta-analysis of TCGA RNA-sequencing level data for *GNA13* expression in normal and tumor tissue from the GEPIA platform (LIHC; liver hepatocellular carcinoma). TPM: transcripts per kilobase per million mapped reads. **d** Kaplan‒Meier curves display the overall survival of patients with low and high *GNA13* expression in the TCGA dataset from the GEPIA platform. **e** Huh7 cell lysates that were transfected with lncRNA-HEIH plasmid, silncRNA-HEIH, miR-194-5p mimic, or miR-194-5p inhibitor were employed for WB. **f** Similar to (**e**), however, Huh7 cells were transfected with the indicated siRNA and miRNA inhibitor. **g** UPF1 was depleted in the indicated cell lines. GNA13 proteins and mRNA were observed by WB and RT‒qPCR, respectively. The level of target mRNA detected by RT‒qPCR was normalized to that of *GAPDH* mRNA. The indicated cell lines transfected with GNA13 plasmid (**h**) or siGNA13 (**i**) were used to measure cell growth over a 96 h period. **j** Suggested model. *p ≤ 0.05; **p ≤ 0.01.

As *lncRNA-HEIH* regulates GNA13 expression by acting as a decoy of miR-194-5p, we examined the expression correlation for GNA13 vs. miR-194-5p and GNA13 vs. lncRNA-HEIH in HCC patients (Fig. 6a, b). TCGA data analysis revealed that the expression of *GNA13* was inversely correlated with the expression of miR-194-5p, while *lncRNA-HEIH* and *GNA13* expression was positively correlated, suggesting that the lncRNA-HEIH/miR-194-5p/GNA13 axis may exist in HCC tissues. These results confirmed that *GNA13* was more highly expressed in HCC tissues than in normal tissues, and patients with low *GNA13* HCC exhibited a longer survival probability than patients with high *GNA13* expression (Fig. 6c, d). We elucidated the regulatory axis by overexpressing *lncRNA-HEIH*, depleting *lncRNA-HEIH*, and introducing a miR-194-5p mimic or miR-194-5p inhibitor (Fig. 6e and Supplementary Fig. 7a–c). WB results indicated that the overexpression of *lncRNA-HEIH* and the miR-194-5p inhibitor increased the level of GNA13. In contrast, the level of GNA13 was downregulated upon depletion of *lncRNA-HEIH* and treatment with the miR-194-5p mimic. Furthermore, the downregulation of GNA13 expression by depletion of *lncRNA-HEIH* was attenuated by the miRNA-194-5p inhibitor (Fig. 6f). UPF1 depletion in HCC cell lines increased the protein and mRNA levels of GNA13, and this effect was reversed by depletion of *lncRNA-HEIH* (Fig. 6g and Supplementary Fig. 7d). In line with this, the expression levels of *UPF1* and *GNA13* in HCC patients were negatively correlated (Supplementary Fig. 7e)^36^. This observation led us to investigate GNA13 regulates HCC growth. Overexpression and depletion of GNA13 in HCC cell lines promoted and suppressed cell growth, respectively, suggesting that GNA13 works as an oncogene (Fig. 6h, i). These observations suggest that increasing *lncRNA-HEIH* by low UPF1 expression in HCC promotes the miR-194-5p decoy function of *lncRNA-HEIH*, thereby increasing GNA13 expression in HCC patients.

## Discussion

UPF1, a key factor in NMD, UPF1-dependent mRNA decay (UMD) and numerous other posttranscriptional regulation pathways, and it regulates the abundance of lncRNAs and protein-coding mRNAs to determine cell fate or maintain cell homeostasis; some UPF1-bound lncRNAs are involved in tumorigenesis and cancer progression^15,37–40^. In this study, we present multiple lines of evidence for the regulation of the UPF1/lncRNA-HEIH/miR-194-5p/GNA13 axis in HCC. The RNA-seq results and public RNA-seq data indicated that UPF1 depletion upregulated *lncRNA-HEIH* expression. Upregulation of *lncRNA-HEIH* accelerated cell growth and tumorigenesis, which was consistent with the increase in HCC cell growth following *UPF1* depletion (Fig. 1, Supplementary Figs. 1–3). Multiple UPF1 CLIP-seq experiments and unwinding of double-stranded RNA by binding RNA suggest that UPF1 may regulate *lncRNA-HEIH* levels after binding^24,41^. We identified the binding sites of UPF1 on lncRNA-HEIH using public CLIP-seq data and biochemical assays. These binding sites were confirmed by competition and mutational assays in UPF1 binding sites (Fig. 2 and Supplementary Fig. 4).

Inhibition of UPF1 dephosphorylation, overexpression of unphosphorylated UPF1 variants and depletion of SMG1 or SMG5 increased the level of *lncRNA-HEIH* (Fig. 3). However, we were unable to determine the specific mechanism by which UPF1 eliminated *lncRNA-HEIH*. *LncRNA-HEIH* regulation requires phosphorylated UPF1 by SMG1, which is expected to interact with SMG5, 6, and 7 in a canonical NMD^42^. However, only SMG5 is required for the stability of *lncRNA-HEIH*. As SMG5 or phosphorylated UPF1 can interact with PNRC2^43,44^, we investigated whether PNRC2 affects *lncRNA-HEIH* stability. However, the depletion of PNRC2 had no effect on the level of *lncRNA-HEIH* (Fig. 3a). Moreover, G3BP1, which facilitates UPF1-mediated RNA decay in highly structured transcripts^45^, was not involved in regulating the stability of *lncRNA-HEIH* (Fig. 3a). Overall, we established that phosphorylated UPF1 may regulate the abundance of *lncRNA-HEIH*.

Using public databases, RNA-seq, and biochemical assays, we also demonstrated that *lncRNA-HEIH* acts as a decoy of miR-194-5p, which is abundant in HCC (Figs. 4, 5 and Supplementary Fig. 5)^46,47^. The expression of *lncRNA-HEIH* was inversely correlated with the expression of miR-194-5p, and overexpression of *lncRNA-HEIH* derepressed putative miR-194-5p targets. Furthermore, it was found that miR-194-5p was enriched with *lncRNA-HEIH* and that miR-194-5p reduced the levels of *lncRNA-HEIH* and GNA13 (Figs. 5, 6 Supplementary Figs. 6, 7), which are involved in the metastatic potential of breast cancer and may serve as a prognostic biomarker for HCC patients^48,49^. Consistently, overexpression and depletion of GNA13 promoted and suppressed the growth of HCC cell lines, respectively. The regulatory UPF1/lncRNA-HEIH/miR-194-3p/GNA13 axis depicted in Fig. 6j aids in understanding how UPF1 contributes to HCC tumorigenesis.

Previous studies reported that the expression of UPF1 in HCC was lower than that in normal tissues^15,21,50^, and this phenotype may activate the lncRNA-HEIH/miR-194-5p/GNA13 axis in HCC. In conclusion, we propose that UPF1 is an HCC repressor that acts by regulating the lncRNA-HEIH/miR-194-5p/GNA13 axis; in addition, it has wide-ranging therapeutic applications and is a promising biomarker for HCC. For instance, specific delivery of siRNA targeting *lncRNA-HEIH* or *GNA13* into HCC would be useful in therapeutic approaches for HCC.

### Supplementary information

Supplementary Information file

## Data Availability

RNA-seq data supporting the results of this study have been deposited in the NCBI GEO database under accession number GSE216667.

## References

[1] Llovet JM (2021). Hepatocellular carcinoma. Nat. Rev. Dis. Primers.

[2] Huang MD (2015). Long non-coding RNA TUG1 is up-regulated in hepatocellular carcinoma and promotes cell growth and apoptosis by epigenetically silencing of KLF2. Mol. Cancer.

[3] Pang Y (2020). Peptide SMIM30 promotes HCC development by inducing SRC/YES1 membrane anchoring and MAPK pathway activation. J. Hepatol..

[4] Li Z (2018). The LINC01138 drives malignancies via activating arginine methyltransferase 5 in hepatocellular carcinoma. Nat. Commun..

[5] Wang Y (2019). A novel lncRNA MCM3AP-AS1 promotes the growth of hepatocellular carcinoma by targeting miR-194-5p/FOXA1 axis. Mol. Cancer.

[6] Yuan JH (2014). A long noncoding RNA activated by TGF-β promotes the invasion-metastasis cascade in hepatocellular carcinoma. Cancer Cell.

[7] Yamashita A, Ohnishi T, Kashima I, Taya Y, Ohno S (2001). Human SMG-1, a novel phosphatidylinositol 3-kinase-related protein kinase, associates with components of the mRNA surveillance complex and is involved in the regulation of nonsense-mediated mRNA decay. Genes Dev..

[8] Okada-Katsuhata Y (2012). N- and C-terminal Upf1 phosphorylations create binding platforms for SMG-6 and SMG-5:SMG-7 during NMD. Nucleic Acids Res..

[9] Ohnishi T (2003). Phosphorylation of hUPF1 induces formation of mRNA surveillance complexes containing hSMG-5 and hSMG-7. Mol. Cell.

[10] Kurosaki T, Popp MW, Maquat LE (2019). Quality and quantity control of gene expression by nonsense-mediated mRNA decay. Nat. Rev. Mol. Cell Biol..

[11] Bühler M, Steiner S, Mohn F, Paillusson A, Mühlemann O (2006). EJC-independent degradation of nonsense immunoglobulin-mu mRNA depends on 3’ UTR length. Nat. Struct. Mol. Biol..

[12] Park J (2019). UPF1/SMG7-dependent microRNA-mediated gene regulation. Nat. Commun..

[13] Hogg JR, Goff SP (2010). Upf1 senses 3’UTR length to potentiate mRNA decay. Cell.

[14] Zünd D, Gruber AR, Zavolan M, Mühlemann O (2013). Translation-dependent displacement of UPF1 from coding sequences causes its enrichment in 3’ UTRs. Nat. Struct. Mol. Biol..

[15] Zhou Y (2019). UPF1 inhibits the hepatocellular carcinoma progression by targeting long non-coding RNA UCA1. Sci. Rep..

[16] Li Y (2019). Long non-coding RNA SNAI3-AS1 promotes the proliferation and metastasis of hepatocellular carcinoma by regulating the UPF1/Smad7 signalling pathway. J. Cell. Mol. Med..

[17] Lee S (2022). UPF1 inhibits hepatocellular carcinoma growth through DUSP1/p53 signal pathway. Biomedicines.

[18] Yang F (2011). Long noncoding RNA high expression in hepatocellular carcinoma facilitates tumor growth through enhancer of zeste homolog 2 in humans. Hepatology.

[19] Shen Q (2020). LncRNA HEIH confers cell sorafenib resistance in hepatocellular carcinoma by regulating miR-98-5p/PI3K/AKT Pathway. Cancer Manag. Res..

[20] Gopalappa R, Suresh B, Ramakrishna S, Kim HH (2018). Paired D10A Cas9 nickases are sometimes more efficient than individual nucleases for gene disruption. Nucleic Acids Res..

[21] Chang L (2016). The human RNA surveillance factor UPF1 regulates tumorigenesis by targeting Smad7 in hepatocellular carcinoma. J. Exp. Clin. Cancer Res..

[22] Li C (2020). The C/D box small nucleolar RNA SNORD52 regulated by Upf1 facilitates Hepatocarcinogenesis by stabilizing CDK1. Theranostics.

[23] Ge Z, Quek BL, Beemon KL, Hogg JR (2016). Polypyrimidine tract binding protein 1 protects mRNAs from recognition by the nonsense-mediated mRNA decay pathway. Elife.

[24] Colombo M, Karousis ED, Bourquin J, Bruggmann R, Mühlemann O (2017). Transcriptome-wide identification of NMD-targeted human mRNAs reveals extensive redundancy between SMG6- and SMG7-mediated degradation pathways. Rna.

[25] Imamachi N, Salam KA, Suzuki Y, Akimitsu N (2017). A GC-rich sequence feature in the 3’ UTR directs UPF1-dependent mRNA decay in mammalian cells. Genome Res..

[26] Durand S, Franks TM, Lykke-Andersen J (2016). Hyperphosphorylation amplifies UPF1 activity to resolve stalls in nonsense-mediated mRNA decay. Nat. Commun..

[27] Cesana M (2011). A long noncoding RNA controls muscle differentiation by functioning as a competing endogenous RNA. Cell.

[28] Yuan JH (2017). The MBNL3 splicing factor promotes hepatocellular carcinoma by increasing PXN expression through the alternative splicing of lncRNA-PXN-AS1. Nat. Cell. Biol..

[29] Zhou W (2017). The lncRNA H19 mediates breast cancer cell plasticity during EMT and MET plasticity by differentially sponging miR-200b/c and let-7b. Sci. Signal..

[30] Statello L, Guo CJ, Chen LL, Huarte M (2021). Gene regulation by long non-coding RNAs and its biological functions. Nat. Rev. Mol. Cell. Biol..

[31] Quinn JJ, Chang HY (2016). Unique features of long non-coding RNA biogenesis and function. Nat. Rev. Genet..

[32] Mas-Ponte D (2017). LncATLAS database for subcellular localization of long noncoding RNAs. Rna.

[33] Rasheed SA (2015). MicroRNA-31 controls G protein alpha-13 (GNA13) expression and cell invasion in breast cancer cells. Mol. Cancer.

[34] Meigs TE, Fields TA, McKee DD, Casey PJ (2001). Interaction of Galpha 12 and Galpha 13 with the cytoplasmic domain of cadherin provides a mechanism for beta -catenin release. Proc. Natl. Acad. Sci. USA.

[35] Rasheed SAK (2018). GNA13 expression promotes drug resistance and tumor-initiating phenotypes in squamous cell cancers. Oncogene.

[36] Liu G (2016). Potential diagnostic and prognostic marker dimethylglycine dehydrogenase (DMGDH) suppresses hepatocellular carcinoma metastasis in vitro and in vivo. Oncotarget.

[37] Li L (2017). The human RNA surveillance factor UPF1 modulates gastric cancer progression by targeting long Non-Coding RNA MALAT1. Cell. Physiol. Biochem..

[38] Shao L (2019). UPF1 regulates the malignant biological behaviors of glioblastoma cells via enhancing the stability of Linc-00313. Cell Death Dis.

[39] He J, Ma X (2021). Interaction Between LncRNA and UPF1 in Tumors. Front. Genet..

[40] Zhang Y (2022). The m(6)A demethylase ALKBH5-mediated upregulation of DDIT4-AS1 maintains pancreatic cancer stemness and suppresses chemosensitivity by activating the mTOR pathway. Mol. Cancer.

[41] Fiorini F, Bagchi D, Le Hir H, Croquette V (2015). Human Upf1 is a highly processive RNA helicase and translocase with RNP remodelling activities. Nat. Commun..

[42] Boehm V (2021). SMG5-SMG7 authorize nonsense-mediated mRNA decay by enabling SMG6 endonucleolytic activity. Nat. Commun..

[43] Nicholson P, Gkratsou A, Josi C, Colombo M, Mühlemann O (2018). Dissecting the functions of SMG5, SMG7, and PNRC2 in nonsense-mediated mRNA decay of human cells. Rna.

[44] Cho H (2013). SMG5-PNRC2 is functionally dominant compared with SMG5-SMG7 in mammalian nonsense-mediated mRNA decay. Nucleic Acids Res..

[45] Fischer JW, Busa VF, Shao Y, Leung AKL (2020). Structure-Mediated RNA Decay by UPF1 and G3BP1. Mol. Cell.

[46] Meng Z (2010). miR-194 is a marker of hepatic epithelial cells and suppresses metastasis of liver cancer cells in mice. Hepatology.

[47] Wang X, He Y, Mackowiak B, Gao B (2021). MicroRNAs as regulators, biomarkers and therapeutic targets in liver diseases. Gut.

[48] Xu Y (2016). High expression of GNA13 is associated with poor prognosis in hepatocellular carcinoma. Sci. Rep..

[49] Yagi H (2011). A synthetic biology approach reveals a CXCR4-G13-Rho signaling axis driving transendothelial migration of metastatic breast cancer cells. Sci. Signal..

[50] Zhang H, You Y, Zhu Z (2017). The human RNA surveillance factor Up-frameshift 1 inhibits hepatic cancer progression by targeting MRP2/ABCC2. Biomed. Pharmacother..

