# Supplementary material for: Role of UPF1 in lncRNA-HEIH regulation for hepatocellular carcinoma therapy
Source: Exp Mol Med. 2024 Feb 1;56(2):344–54. doi: 10.1038/s12276-024-01158-6 (PMC10907594; doi:10.1038/s12276-024-01158-6)
Supplement: Supplementary file 1 — Supplementary Information file [file 12276_2024_1158_MOESM1_ESM.pdf]

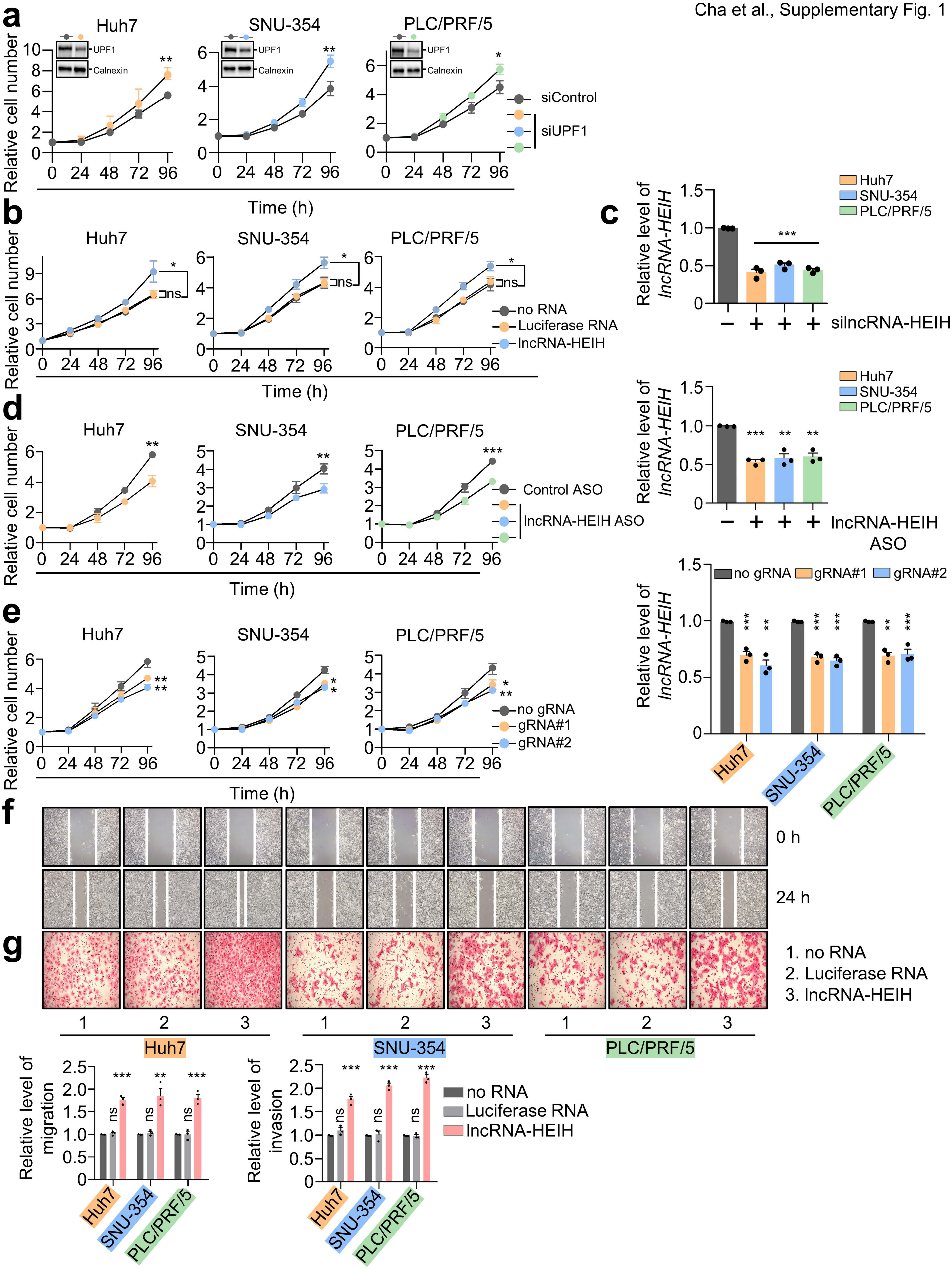
a
Huh7
SNU-354
PLC/PRF/5
UPF1
Calnexin
Relative cell number
Time (h)
siControl
siUPF1
b
no RNA
Luciferase RNA
lncRNA-HEIH
ns
c
Relative level of lncRNA-HEIH
Huh7
SNU-354
PLC/PRF/5
silncRNA-HEIH
lncRNA-HEIH ASO
no gRNA
gRNA#1
gRNA#2
d
Control ASO
lncRNA-HEIH ASO
e
f
0 h
24 h
g
1. no RNA
2. Luciferase RNA
3. lncRNA-HEIH
Relative level of migration
Relative level of invasion
no RNA
Luciferase RNA
lncRNA-HEIH

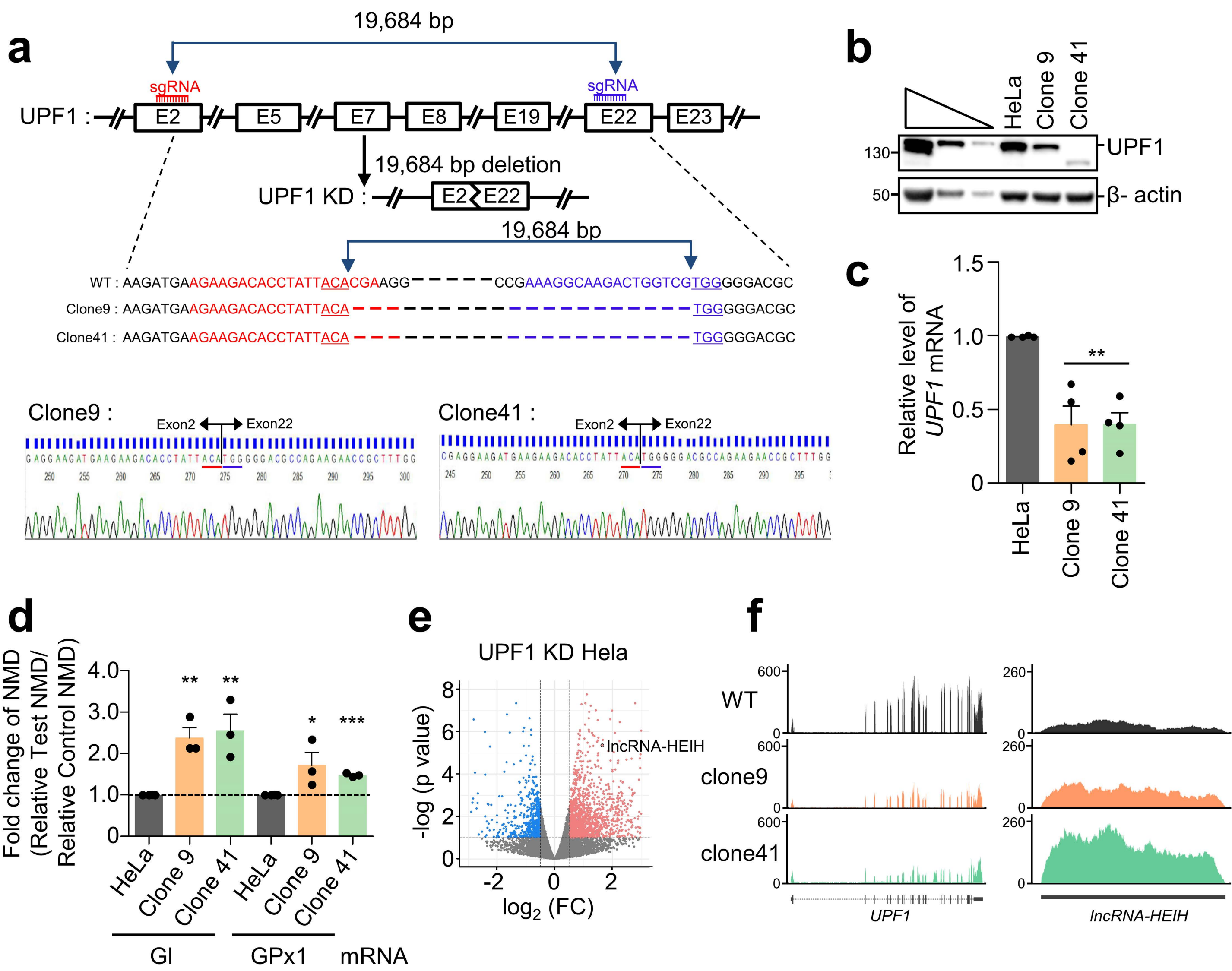
a
19,684 bp
sgRNA
sgRNA
UPF1 :
E2
E5
E7
E8
E19
E22
E23
19,684 bp deletion
UPF1 KD :
E2
E22
19,684 bp
WT : AAGATGAAGAAGACACCTATTACACGAAGG CCGAAAGGCAAGACTGGTCGTGGGGGACGC
Clone9 : AAGATGAAGAAGACACCTATTACA TGGGGGACGC
Clone41 : AAGATGAAGAAGACACCTATTACA TGGGGGACGC
Clone9 :
Exon2
Exon22
Clone41 :
Exon2
Exon22
b
HeLa
Clone 9
Clone 41
130
UPF1
50
β- actin
c
Relative level of UPF1 mRNA
1.5
1.0
0.5
0
**
HeLa
Clone 9
Clone 41
d
Fold change of NMD (Relative Test NMD/ Relative Control NMD)
4.0
3.0
2.0
1.0
0
**
**
*
***
HeLa
Clone 9
Clone 41
HeLa
Clone 9
Clone 41
GI
GPx1
mRNA
e
UPF1 KD Hela
-log (p value)
8
6
4
2
0
-2
0
2
log2 (FC)
lncRNA-HEIH
f
WT
clone9
clone41
600
0
260
UPF1
lncRNA-HEIH

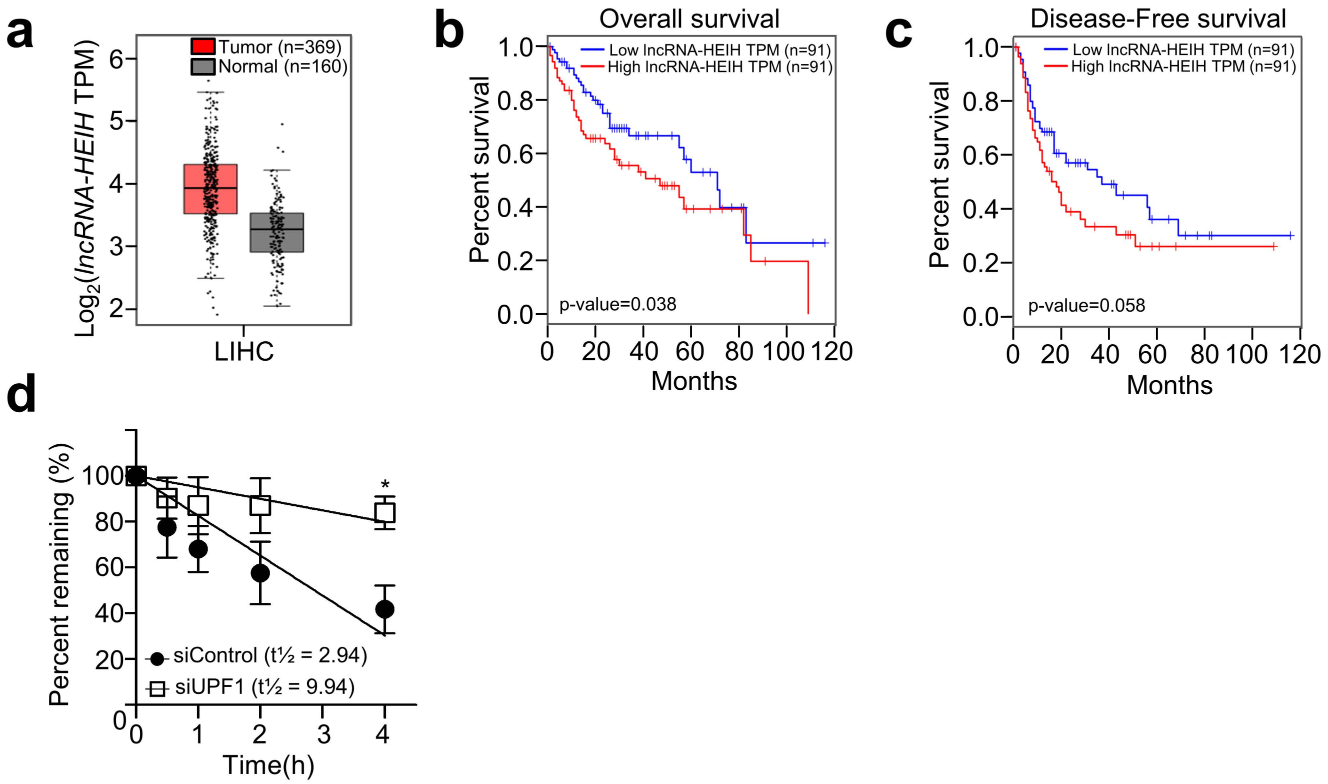
a
Tumor (n=369)
Normal (n=160)
Log2(lncRNA-HEIH TPM)
LIHC
b
Overall survival
Low lncRNA-HEIH TPM (n=91)
High lncRNA-HEIH TPM (n=91)
Percent survival
p-value=0.038
Months
c
Disease-Free survival
Low lncRNA-HEIH TPM (n=91)
High lncRNA-HEIH TPM (n=91)
Percent survival
p-value=0.058
Months
d
Percent remaining (%)
siControl (t½ = 2.94)
siUPF1 (t½ = 9.94)
Time(h)

a

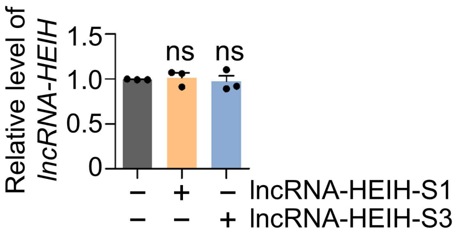
Relative level of
lncRNA-HEIH
1.5
1.0
0.5
0
ns
ns
– + – lncRNA-HEIH-S1
– – + lncRNA-HEIH-S3

b

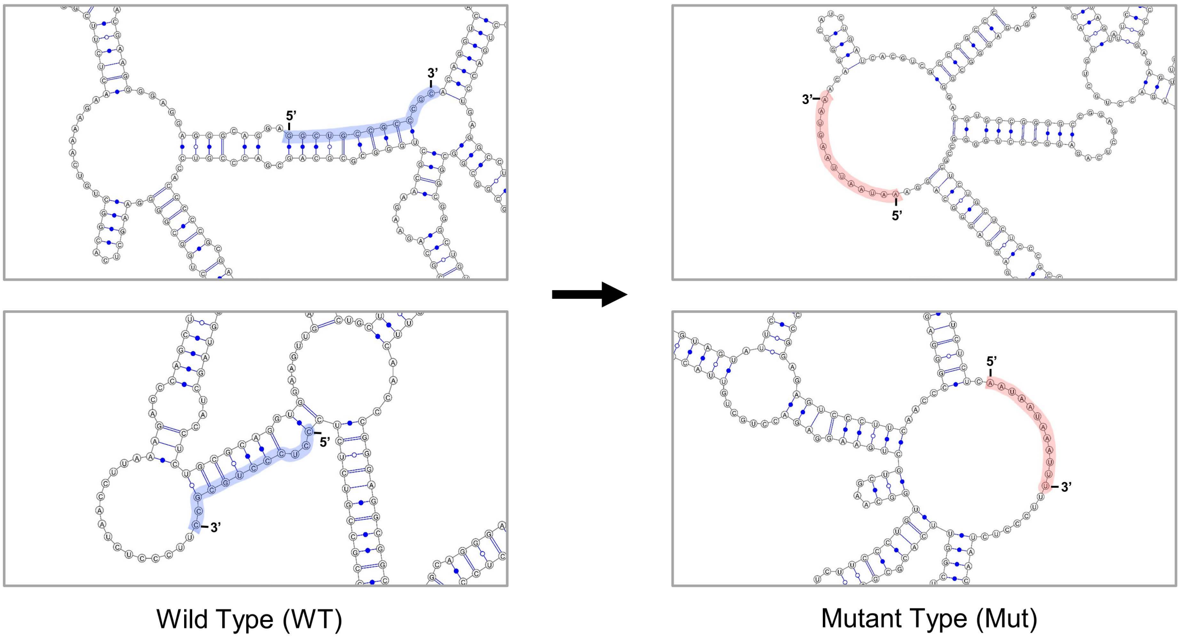
5'
3'
Wild Type (WT)
Mutant Type (Mut)

c

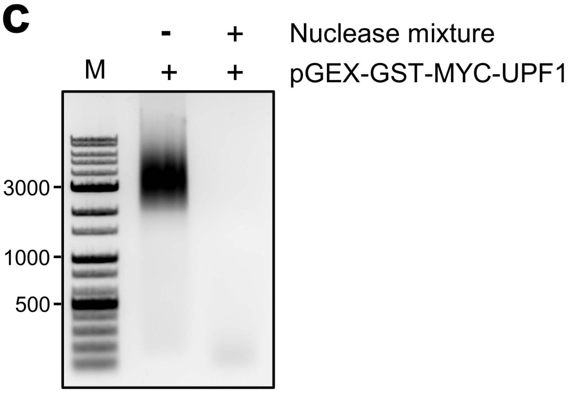
- + Nuclease mixture
M + + pGEX-GST-MYC-UPF1
3000
1000
500

d

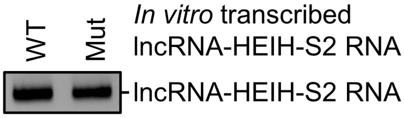
WT
Mut
In vitro transcribed
lncRNA-HEIH-S2 RNA
lncRNA-HEIH-S2 RNA

Cha et al., Supplementary Fig. 5

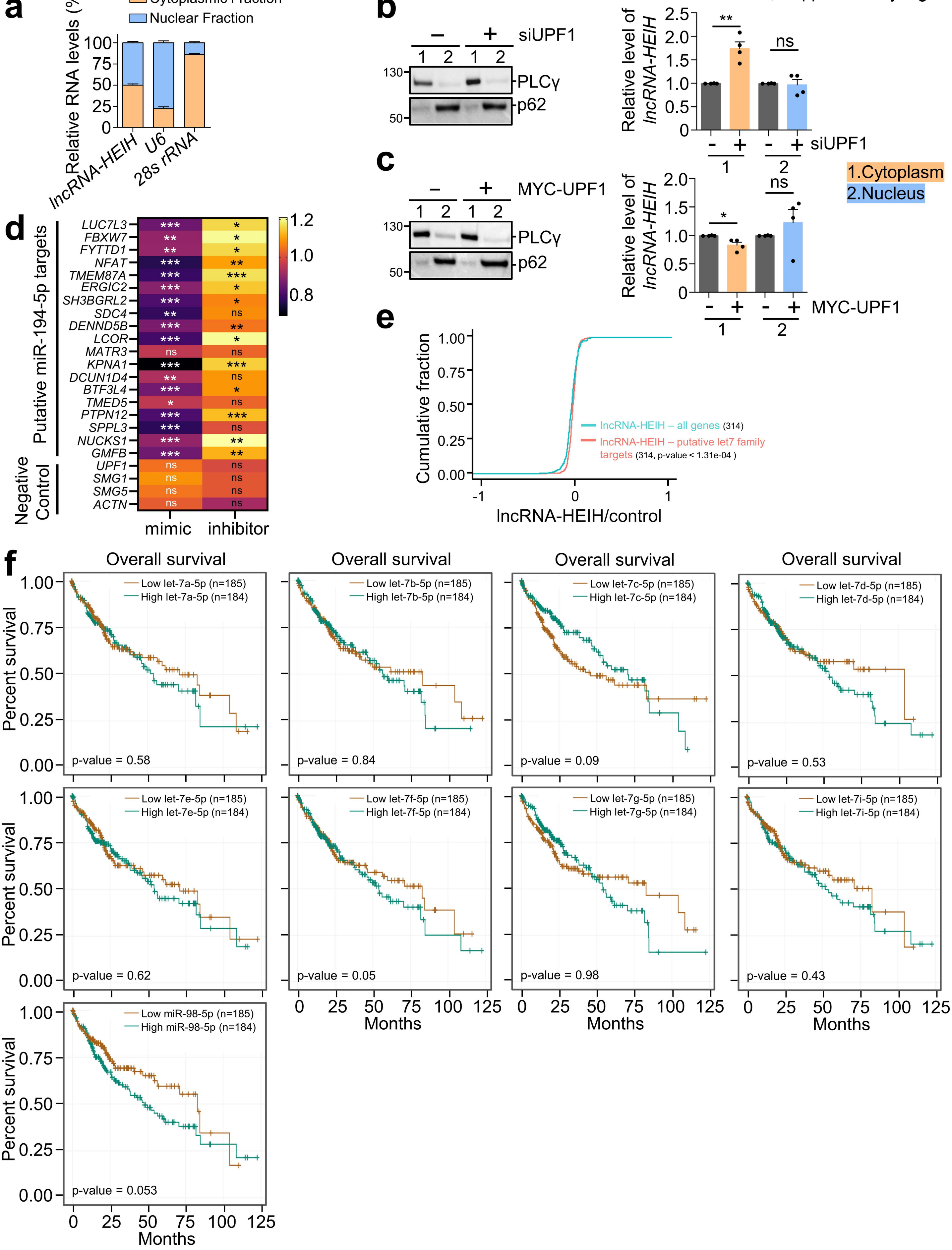

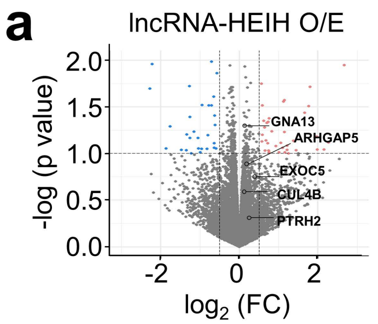
a
lncRNA-HEIH O/E
-log (p value)
2.0
1.5
1.0
0.5
0.0
-2
0
2
log2 (FC)
GNA13
ARHGAP5
EXOC5
CUL4B
PTRH2

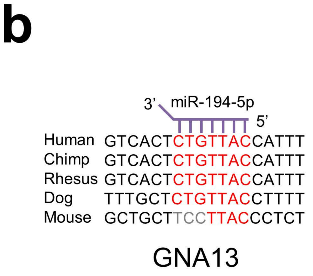
b
3'
miR-194-5p
5'
Human GTCACTCTGTTACCATTT
Chimp GTCACTCTGTTACCATTT
Rhesus GTCACTCTGTTACCATTT
Dog TTTGCTCTGTTACCTTTT
Mouse GCTGCTTCCTTACCCTCT
GNA13

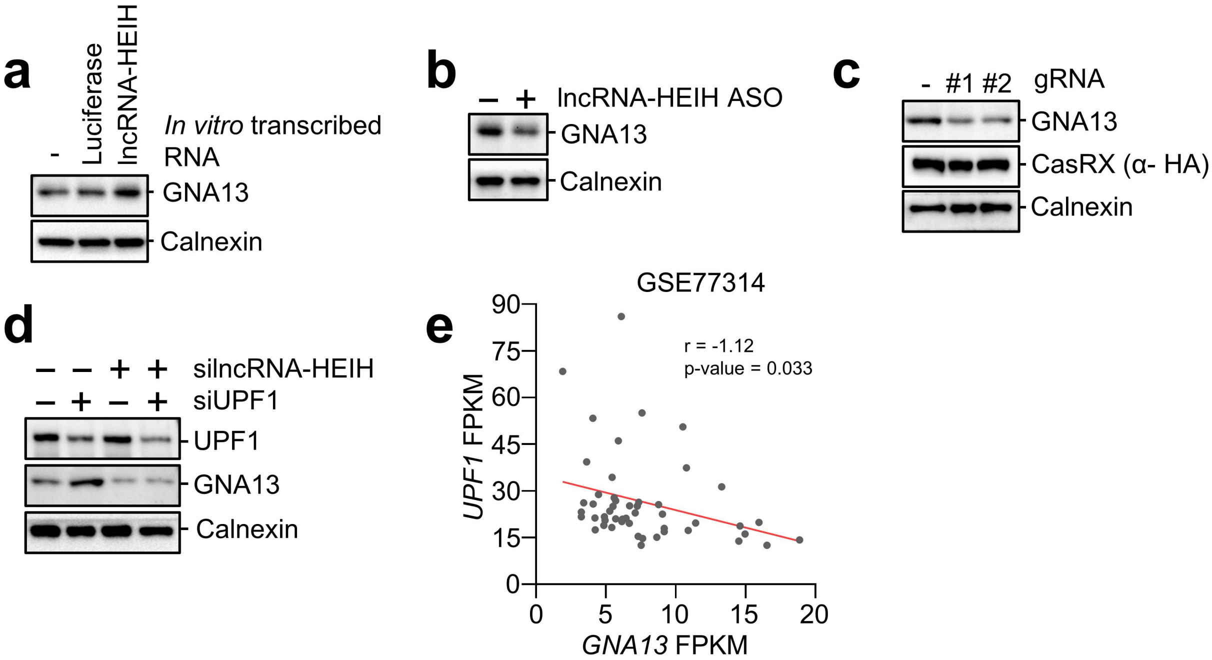
a
-
Luciferase
lncRNA-HEIH
In vitro transcribed RNA
GNA13
Calnexin
b
– +
lncRNA-HEIH ASO
GNA13
Calnexin
c
- #1 #2
gRNA
GNA13
CasRX (α- HA)
Calnexin
d
– – + +
silncRNA-HEIH
– + – +
siUPF1
UPF1
GNA13
Calnexin
e
GSE77314
r = -1.12
p-value = 0.033
UPF1 FPKM
GNA13 FPKM
0
15
30
45
60
75
90
0
5
10
15
20

Supplementary Figure Legends

Supplementary Fig. 1 a Depletion of UPF1 augmented HCC cell growth. HCC cells where UPF1 was depleted were employed for cell growth by cell counting over a period of 96 h. WB was performed to evaluate UPF1 expression. **b** (Related to Fig. 1e) Similar to Fig. 1e, however, *in vitro* transcribed *lncRNA-HEIH* or *Firefly luciferase* RNA was transfected to the indicated HCC cells. **c** RT-qPCR was performed to evaluate the level of *lncRNA-HEIH* in Fig. 1f. **d, e** (Related to Fig. 1f) ASO (d) and CasRX (e) were employed to deplete *lncRNA-HEIH*. RT-qPCR was performed to evaluate the level of *lncRNA-HEIH*. **f**, **g** (Related to Fig. 1h and 1i) Similar to Supplementary Fig. 1b, however, cell migration assay (f) and invasion assay (g) were performed using the indicated HCC cell lines that were transfected with *lncRNA-HEIH* or *Firefly luciferase* RNA. Relative levels of migration and invasion were quantified. The relative level of mRNA was normalized to that of *GAPDH* mRNA in (c, d, and e). * $p \leq 0.05$; ** $p \leq 0.01$; *** $p \leq 0.001$; ns: not significant.

Supplementary Fig. 2 UPF1 knockdown cell lines exhibits high expression of *lncRNA-HEIH*. **a** Representation of UPF1 KD HeLa cell lines by CRISPR/Cas9. Two guide RNAs targeting exon2 and exon22 were indicated. Cells were selected by puromycin. **b, c** WB (b) and RT-qPCR (c) were performed to determine the reduced UPF1 expression in two clones, clone 9 and clone 41. The relative level of *UPF1* mRNA was normalized to that of *GAPDH* mRNA. **d** The relative amounts of NMD reporter constructs (Gl and GPx1) transfected into the indicated cell lines were assessed by RT-qPCR. Relative NMD is expressed as the ratio of the level of Ter mRNA to the level of Norm mRNA, each of which was normalized to *MUP* reference mRNA. **e** Volcano plots showing the log2-fold change in expression between UPF1 KD HeLa cell lines (clones 9 and clone 41) and

normal HeLa cell lines. **f** Integrative genomics viewer (IGV) displays the increased level of *lncRNA-HEIH* in the indicated cell lines from RNA-sequencing in (E). * $p \leq 0.05$; ** $p \leq 0.01$; *** $p \leq 0.001$.

Supplementary Fig. 3 *LncRNA-HEIH* is upregulated in LIHC and related to poor prognosis. **a** Differential level of *lncRNA-HEIH* in liver hepatocellular carcinoma (LIHC) tissues and normal liver tissues analyzed through the TCGA dataset from GEPIA platform. **b, c** Overall survival (b) or disease-free survival (c) in LIHC patients expressing high or low levels of *lncRNA-HEIH* from GEPIA platform. **d** Huh7 cells transfected with siUPF1 or control siRNA were treated with 100 μg/ml DRB for 4 h. The level of *lncRNA-HEIH* was measured at the indicated time point by RT-qPCR. The relative level of mRNA was normalized to that of *GAPDH* mRNA. * $p \leq 0.05$.

Supplementary Fig. 4 a (Related to Fig. 2d) The relative *lncRNA-HEIH* levels were measured by RT-qPCR after transfection of the S1 or S3 region of lncRNA-HEIH (lncRNA-HEIH-S1 or -S3) in Huh7 cells. **b** Schematic representation of the predictive RNA structure of UPF1 binding sites (blue shade, left panel) and mutated UPF1 binding sites (red shade, right panel) in lncRNA-HEIH by ViennaRNA RNA fold. **c** (Related to Fig. 2j) To determine whether the nuclease mixture (RNase, DNase, and MNase) completely removed the nucleic acids, cell lysates that were transfected with pGEX-GST-MYC-UPF1 were treated with nuclease mixture and were loaded onto an agarose gel. The DNA and RNA were visualized using EtBr staining. **d** (Related to Fig. 2j) *In vitro* transcribed biotinylated RNA was loaded onto an agarose gel to determine RNA quality. The RNA was visualized using EtBr staining. The relative level of mRNA was normalized to that of *GAPDH*

mRNA (a). ns: not significant.

Supplementary Fig. 5 **a** Subcellular localization of *lncRNA-HEIH, snU6 RNA*, and *28S rRNA*. Huh7 cells were fractionated and RT-qPCR was performed to evaluate the levels of RNA. Cytoplasmic and nuclear RNA were spiked with *in vitro* transcribed *Firefly luciferase* RNA. The relative level of the indicated RNA was normalized to that of *Firefly luciferase* RNA. **b, c** Subcellular localization of *lncRNA-HEIH* in UPF1-depleted (b) or UPF1-overexpressing (c) Huh7 cells. WB was performed to determine cell fractionation. RT-qPCR was performed to evaluate the relative level of *lncRNA-HEIH* in the nucleus and cytoplasm. Nuclear and cytoplasmic *lncRNA-HEIH* were normalized to *snU6 RNA* and *GAPDH* mRNA, respectively. **d** (Related to Fig. 4d) Relative levels of miR-194-5p targets by RT-qPCR were presented as heat-maps upon miR-194-5p mimic or inhibitor treatment in Huh7. The relative level of mRNA was normalized to that of *GAPDH* mRNA. **e** Cumulative log2 fold changes in expression are shown as CDF plots using the putative let-7 targets, which have over 10 FPKM in RNA-seq using *lncRNA-HEIH*-overexpressing Huh7 cells. **f** Kaplan-Meier curves displays overall survival of patients with low vs high expressions of the indicated miRNAs in TCGA database from starBase V2.0 platform. * $p \leq 0.05$; ** $p \leq 0.01$; *** $p \leq 0.001$; ns: not significant.

Supplementary Fig. 6 **a** Volcano plots showing the log2-fold change in expression between *lncRNA-HEIH* overexpressing Huh7 cells and control Huh7 cells. **b** Conservation of the putative miR-194-5p binding sequences in GNA13 3'UTR through mammals.

Supplementary Fig. 7 a Huh7 cell were transfected with *in vitro* transcribed *Firefly luciferase* RNA or *lncRNA-HEIH*. **b, c** *LncRNA-HEIH* was depleted by ASO (b) and CasRX (c) targeting lncRNA-HEIH in Huh7, respectively. Two guideRNA (gRNA#1 and #2) were employed. **d** Huh7 cells were transfected the indicated siRNA. WB was performed to evaluate the GNA13 expression in (a, b, c, and d). **e** The expression of *UPF1* and *GNA13* in HCC was analyzed using public data under accession number (GSE77314).

Supplementary Table 1. siRNA used in this study

siRNA name	Sense (5' to 3')
UPF1-CDS	CCAAGAUGCAGUUCCGCUCCAUU
UPF1-3'UTR	GCUUAGUCCAUCAGCAUCUUAUUCU
SMG7-1	AACAGCACAGUCUACAAGCCA
SMG7-2	GCAAGAAACAUCUGUGAUA
SMG1-CDS	AAGUGUAUGUGCGCCAAAGUAUUUG
SMG1-3'UTR	GGAAGAUUUGAUGCAUUCAGAUUCU
PNRC2	AGUUGGAAUUCUAGCUUAU
SMG6-CDS-1	AAGCCAGUGAUACAGCGAAUU
SMG6-CDS-2	GGGUCACAGUGCUGAAGUA
SMG5-CDS-1	AAGUCUUCCUGGACUGGCUUC
SMG5-CDS-2	GAAGGAAAUUGGUUGAUAC
G3BP1-1	AAACCCACCAAAGACCUCAUCUUGG
G3BP1-2	UAAUUUCCCACCACUGUUAAUGCGC
lncRNA-HEIH-1	AAAAAAAAAAAACAUGGCCGAAAC
lncRNA-HEIH-2	GCAGUAACAGAGUCAACAAGACAAU
GNA13 -1	GGCAUCCAUGAAUACGACUUUGAAA
GNA13 -2	UAGCAGUUUACAACCAGAAUUAGAA

Supplementary Table 2. Cloning primers used in this study

Primer name	Sequence (5' to 3')
HEIH-EcoRI-F	ATAGAATTCGTCCCCGCCCCCTGTT
HEIH-XbaI-R	GCGTCTAGACAAGGTTGGAAAATCCC
HEIH-S2-EcoRI-F	GCGGAATTCTCCACAGCCCAAAGCCA
HEIH-S2-XbaI-R	GCGTCTAGATAAAAGGAGCTCCCTCCC
UPF1-bs-WT-XbaI-F	ATATCTAGAGCACGAAGGGGAGGAGGGC
UPF1-bs-WT-NotI-R	ATAGCGGCCGCCTTTAAGGTTAGAGGGAAG
UPF1-bs-Mut-F	GCACGAAGGGGAGGAGGGCAGGAAATAATTAAGTAAACAGGT CATCTGATCACGTCGCCCGCCCTAGTCTGC
UPF1-bs-Mut-R	GATCACGTCGCCCGCCCTAGTCTGCTTTTGTGAATCTCCACTTTG TTCAACCCCCACCCGCCGTCTCTCAATAATAAATTTTTCCCTCTA ACCTTAAAG

GNA13-3'UTR-5'	GAAATCATGCCTGTAAAGCCCAAACATTTGTAACAAACTCCCTAATAAATTTAG AGAAAGTCACT
GNA13-3'UTR WT-3'	CAAGTGAAAAAGACATGAGCAAAACTTCTGGTCTTAATTTCTCAAATAAAACTAAAATAAATGAAATGGTAACAGAGTGACTTTCTCTAAATTTATTAGG
GNA13-3'UTR-F	CTAGTTGTTTAAACGGAAATCATGCCTGTAA
GNA13-3'UTR WT-R	ATGCCTGCACTCTAGCAAGTGAAAAAGACA
GNA13-3'UTR-Mut-3'	CAAGTGAAAAAGACATGAGCAAAACTTCTGGTCTTAATTTCTCAAATAAAACTAAAATAAATGAAATGGATTGAGAGTGACTTTCTCTAAATTTATTAGG
GNA13-3'UTR-Mut-R	ATGCCTGCACTCTAGCAAGTGAAAAAGACA
HEIH-miR-194-5p-5'	CTCTGCGCAGGTGGAAGTTGAGTTCGGTTCACGCGGGACCCTCTTCCCTGTGGC AAGCTGCTGAAGGAGACC
HEIH-miR-194-5p-WT-3'	GCGAGGGCGGAATACTACCTTCCAGCTGTCTGAGATTAAGCAGAACAGCAGCTAAAGCAGTAACAGCAGGTCTCCTTCAGCAGCTTGC
HEIH-miR-194-5p-F	CTAGTTGTTTAAACGCTCTGCGCAGGTGGAAG
HEIH-miR-194-5p-WT-R	ATGCCTGCACTCTAGGCGAGGGCGGAATACT
HEIH-miR-194-5p-Mut-3'	GCGAGGGCGGAATACTACCTTCCAGCTGTCTGAGATTAAGCAGAACAGCAGCTAAAGCAGATTGAGCAGGTCTCCTTCAGCAGCTTGC
HEIH-miR-194-5p-Mut-R	ATGCCTGCACTCTAGGCGAGGGCGGAATACT
**Fragment1** HEIH-UPF1-bs-Mut-fragment1-F	TGTGGTGGAATTCTGCAGATGTCCCCGCCCCCTGCTG
**Fragment1** HEIH-UPF1-bs-Mut-fragment1-R	TGCCCTCCTCCCCTTCGTGCGCCGG
**Fragment2** HEIH-UPF1-bs-Mut-fragment2-F	GCACGAAGGGGAGGAGG
**Fragment2** HEIH-UPF1-bs-Mut-fragment2-R	CTTTAAGGTTAGAGGGAAAAATTTA
**Fragment3** HEIH-UPF1-bs-Mut-fragment3-F	TTTTCCCTCTAACCTTAAAGACCCAGCTACCTCTACGCAAATGGT
**Fragment3** HEIH-UPF1-bs-Mut-fragment3-R	CGGCCGCCACTGTGCTGGATCAAGGTTGGAAAATCCCA
HEIH-S2-UPF1-bs-Mut-HindIII-F	GCGAAGCTTTCCACAGCCCAAAGCCAC
HEIH-S2-UPF1-bs-Mut-NotI-R	ATAGCGGCCGCTAAAAGGAGCTCCCTC
GNA13-F	CAG TGT GGT GGA ATT CAT GGC GGA CTT CCT GCC GT
GNA13-R	CTT TGT AGT CCT CGA GTC ACT GTA GCA TAA GCT GC
**Fragment** gRNA-#1	GTCGGGGTTTGAAACTCTTCCCTTGATCTAAGAAGGTTTTTTTTGAATTCTG
**Fragment** gRNA-#2	GTCGGGGTTTGAAACGCAGTAACAGAGTCAACAAGACATTTTTTTGAATTCTG
HEIH-S1-XbaI-R	GCGTCTAGAAACGCTGCCTCTGTGGAA

HEIH-S3-EcoRI-F	GCGGAATTCTCTTGTTACCTACTCCCC
luciferase-F	TTGGTACCGAGCTCGATGGAAGATGCCAAAAAC
luciferase-R	CCGTTCTAGCGGCACATTGACGTCTATAGGTCG

Supplementary Table 3. Antisense or sense oligo used in this study

Primer name	Sequence (5’ to 3’)
Sense lncRNA-HEIH	GCAGTAACAGAGTCAACAAGACAAT
Antisense lncRNA-HEIH-1	ATTGTCTTGTTGACTCTGTTACTGC
Antisense lncRNA-HEIH-2	CTTCCCTTGATCTAAGAAG

Supplementary Table 4. qPCR primers used in this study

primer name	Forward (5’ to 3’)	Reverse (3’ to 5’)
lncRNA-HEIH	GCGCATAGGCCGGTTCTAAT	GCTTCCTGGTTTCGGCCATGTTT
Gl	TGCACGTGGATCCTGAGAACTTCA	ACCATTGTTCACAGGCAAGAGCAG
GPx1	CGGTTTCCCGTGCAATCAGTTCGG	TCACCATTCACCTCGCACTTCTCA
MUP	CTGATGGGGCTCTATG	TCCTGGTGAGAAGTCTCC
MAP3K14	GGCCCGTGTGTGTTGGAAGGG	GGTTCAGACATTGCAAGGGG
SMG5	CCCGAAGCAAAAGTCCTCCA	TCACGCAGCTTGTTCCTCAG
PEA15	TTAGGAACCGGGGACTCAGG	GCGGAGGCCATTCTATCCAA
MALAT1	TTCGTTTGCCTCAGACAGGT	ACTGAAGCCCACAGGAACAA
BAG1	AAGATGGTTGCCGGGTCATG	TGTTCTGCTCCACTGTGTCAC
TBL2	GCAGTCATTTACCACATGC	TATTGTTTCTGCTTCTTGGAT
GAPDH	CAAGATCATCAGCAATGCC	CTGTGGTCATGAGTCCTTCC
Actin	ACAAGCTGAGGAAGGACGAC	GGCATGGTAGAAGCTGGACA
UPF1	GAGGCCGACTACGACAAGAA	CATGAGCCGCATGTCAGAGT
SMG1	TCCTCGGATCGAGAGTGAATC	TCTCCCTGACTGGCATTGTC
SMG6	GCCGGGAGCAGAGAAAACA	ACAGCAGAGCAATCTCGGTC
SMG7	GGCAGGCAGAAGTCCTGAAG	AGGCGTGATTCCAGAGATCC
FLuc	CATGGATAGCAAGACCGACTAC	TCAGGGCGATGGTTTTGTC
RLuc	AGATCATGCGGAAACTGGAG	CGAAGGTAGGCGTTGTAGTTG
miR-194-5p	TGTAACAGCAACTCCATGTG	GTGCAGGGTCCGAGGT
GNA13	TCCACCTTCCTGAAGCAGATGC	GCTTCTCTCGAGCATCAACCAG
Exoc5	CTTCAGTAATCCAGAAACAGTCCT	TGCTCTGCATCGGACTTCCTAC
CUL4B	GAAGCTACAGATGAAGAACTTGAG	GCACTCTTTCCGACTAACAGGC
ARHGAP5	CTCCAGGATCTGGTTACAGCTG	GTCTTGGTCAGTTTTATTCCCGC
PTRH2	GCTAAAAGACCGTGCCAGTGATC	TGGCACCTTCATTACCAGCACG
exo HEIH	CTAGCACACCCATTGTGGAG	CTGATCAGCGAGCTCTAGCA

Exo HEIH S2	AACGTCTGAAATGGAGGCCC	CTGATCAGCGAGCTCTAGCA
PNRC2	CAAAGTTGGAATTCTAGCTTATCAG	GGGAAGAACACTTGGTGATGGC
G3BP1	AGCCTGTTCAGAAAGTCCTTAGC	CGAAGGCGATTATCTCGTCGGT
sqPCR lncRNA-HEIH	GCGGAATTCATGGCCGAAACCAGGAAGC	CTGCCCTCCTCCCCTTC
sqPCR GNA13	TCCACCTTCCTGAAGCAGATGC	GGATGCCTTTGGTGGGTCTTC
LUC7L3	CACAGGAGCAAAAGTCGGGACA	GTGTCTTCACTCTGCTTTTCTCG
FBXW7	GTTTGGTCAGCAGTCACAGGCA	CCACACTTTGAGTGTCCGATCTG
FYTTD1	CCAGCAATTCAGGATGAGAGTGC	CCAGTCGTTTTCCTAGCTGCAAG
NFAT	CCTAATGCCCTGATGACTCCAC	GTTTGCTGAGTTGATCCAACAGAC
TMEM87A	GATTGGTGCTGTCATCTTCCTGG	TTCGAGCCAGTGAGCGTTTCAC
ERGIC2	TGAGGAGCACATGCCATTCTGG	GGATCCAAGTCTGAAACGACAGC
SH3BGRL2	GAAGAACAGAGGCAATGGATGTAC	GACTGTGTTGCTTTCCTTGGATTC
SDC4	GAGTGAGGATGTGTCCAACAAGG	GGTACATGAGCAGTAGGATCAGG
DENND5B	CAGTCCGATGTCTTGGCAACAG	TCTCCCTCAAGTCGTTGTCCAG
LCOR	AAGTCCATGTGCTGGCAGCACT	ATCACCACTCCGAAGTCCGTCT
MATR3	CAGCAGTCTACAAATCCAGCACC	CTGCATGTGTCTAGGTCCTTGC
KPNA1	TTCCAAAAGCCCAGAGCAACAGC	CCACTACTCCTGGTGTGCTGAT
DCUN1D4	AGAAGACCTGCCTCTGGAGATG	AGAACCATTCCAAGCACCTTTTAC
BTF3L4	GCTCGCAGAAAGAAGAAGGTGG	GGATTGTTGAAATGAATAACTGTCC
TMED5	AGAAGGAGTGCTTCTACCAGCC	CTAAGGTTTTGCCTTCTGGAGAG
PTPN12	CAGCTCCATAGAGCCTGAAAAAC	GTCTGTCATGCTCGTCCTCATC
SPPL3	GTCTGTCATGCTCGTCCTCATC	AGAAGCAGGCAGGAGACCTTGA
NUCKS1	GACGATAGTGACTATGGCAGTTC	CCTTTCACTGGACTTGGCGTCA
GMFB	CGCAAAGAAACGAACAACGCTGC	CGAGGTTGTCGTTCAGGTAGTTC
U6	TCGCTTCGGCAGCACATATAC	TGCGTGTCATCCTTGCGCAG
28S rRNA	GAAAGCGGGGCCTCACGATCCTT	TGGTAGCTTCGCCCCATTGGCTC

Supplementary Table 5. guideRNA sequence used in this study

Primer name	Sequence (5' to 3')
guideRNA-#1	TCTTCCCTTGATCTAAGAAGGTT
guideRNA-#2	GCAGTAACAGAGTCAACAAGACA
